# Boost of innate immunity cytokines as biomarkers of response to extracorporeal photopheresis in patients with leukaemic cutaneous T-cell lymphoma

**DOI:** 10.1093/bjd/ljad220

**Published:** 2023-07-06

**Authors:** Yi-Chien Tsai, Tanja Schlaepfer, Desislava Ignatova, Yun-Tsan Chang, Alan Valaperti, Boyko Amarov, Gabriela Blanchard, Kevin Pehr, Maya Vonow-Eisenring, Mirjana Urosevic-Maiwald, Wolfram Hoetzenecker, Steve Pascolo, Christoph Iselin, Christina Fassnacht, Florentia Dimitriou, Malgorzata Bobrowicz, Emmanuella Guenova

**Affiliations:** Department of Dermatology, Lausanne University Hospital (CHUV), and Faculty of Biology and Medicine, University of Lausanne, Lausanne, Switzerland; Departments of Dermatology; Departments of Dermatology; Department of Dermatology, Lausanne University Hospital (CHUV), and Faculty of Biology and Medicine, University of Lausanne, Lausanne, Switzerland; Immunology, University Hospital Zürich, Switzerland; Institute of Statistics and Econometrics, Faculty of Economics and Business Administration, Sofia University ‘St Kliment Ohridski’, Sofia, Bulgaria; Department of Dermatology, Lausanne University Hospital (CHUV), and Faculty of Biology and Medicine, University of Lausanne, Lausanne, Switzerland; Division of Dermatology, McGill University, Jewish General Hospital, and Lady Davis Institute for Medical Research, Montreal, Canada; Immunology, University Hospital Zürich, Switzerland; Hautärzte-Zentrum am Zürisse, Zürich, Switzerland; Department of Dermatology, University Hospital Linz, Linz, Austria; Departments of Dermatology; Department of Dermatology, Lausanne University Hospital (CHUV), and Faculty of Biology and Medicine, University of Lausanne, Lausanne, Switzerland; Departments of Dermatology; Departments of Dermatology; Department of Immunology, Medical University of Warsaw, Warsaw, Poland; Department of Dermatology, Lausanne University Hospital (CHUV), and Faculty of Biology and Medicine, University of Lausanne, Lausanne, Switzerland; Departments of Dermatology

## Abstract

**Background:**

Extracorporeal photopheresis (ECP) has emerged as a systemic first-line immunomodulatory therapy in leukaemic cutaneous T-cell lymphoma (L-CTCL) and is now beginning to be utilized in other T-cell-mediated diseases. Although ECP has been used for nearly 30 years, its mechanisms of action are not sufficiently understood, and biomarkers for response are scarce.

**Objectives:**

We aimed to investigate the immunomodulatory effects of ECP on cytokine secretion patterns in patients with L-CTCL, to help elucidate its mechanism of action.

**Methods:**

A total of 25 patients with L-CTCL and 15 healthy donors (HDs) were enrolled in this retrospective cohort study. Concentrations of 22 cytokines were simultaneously quantified by using multiplex bead-based immunoassays. Neoplastic cells in patients’ blood were evaluated by flow cytometry.

**Results:**

Firstly, we observed a distinct cytokine profile pattern difference between L-CTCLs and HDs. There was a significant loss of tumour necrosis factor (TNF)-α, and significant increase of interleukins (IL)-9, IL-12 and IL-13 in the sera of patients with L-CTCL compared with HDs. Secondly, patients with L-CTCL who received ECP were classified as treatment responders and nonresponders according to the quantitative reduction of malignant burden in their blood. We evaluated cytokine levels in culture supernatants from patients’ peripheral blood mononuclear cells (PBMCs) at baseline and 27 weeks after ECP initiation. Strikingly, PBMCs purified from ECP responders released statistically higher concentrations of innate immune cytokines IL-1α, IL-1β, granulocyte–macrophage colony-stimulating factor (GM-CSF) and TNF-α in comparison with ECP nonresponders. In parallel, responders showed clearance of erythema, reduction of malignant clonal T cells in the blood, and a potent boost of relevant innate immune cytokines in individual patients with L-CTCL.

**Conclusions:**

Taken together, our results demonstrate that ECP stimulates the innate immune network, and facilitates redirection of the tumour-biased immunosuppressive microenvironment towards proactive antitumour immune responses. The alterations of IL-1α, IL-1β, GM-CSF and TNF-α can be used as biomarkers of response to ECP in patients with L-CTCL.

## Teaser

Extracorporeal photopheresis (ECP) has emerged as a systemic first-line immunomodulatory therapy in leukaemic cutaneous T-cell lymphoma (L-CTCL). However, the predictive biomarkers for ECP efficacy are still missing. We demonstrated that patients with L-CTCL carry a distinct cytokine secretion pattern compared with healthy individuals. Furthermore, effective ECP therapy facilitates recovery of the immune profile in ECP responders, and stimulates the release of innate immune cytokines IL-1α, IL-1β, GM-CSF and TNF-α from patients’ PBMCs. Our results support that effective ECP activates the innate immune network and in favour of proactive antitumour immune responses, and we believe that the elevated secretion of IL-1α, IL-1β, GM-CSF and TNF-α are promising biomarkers for efficient ECP therapy.

## Graphical Abstract

**Graphical Abstract:**
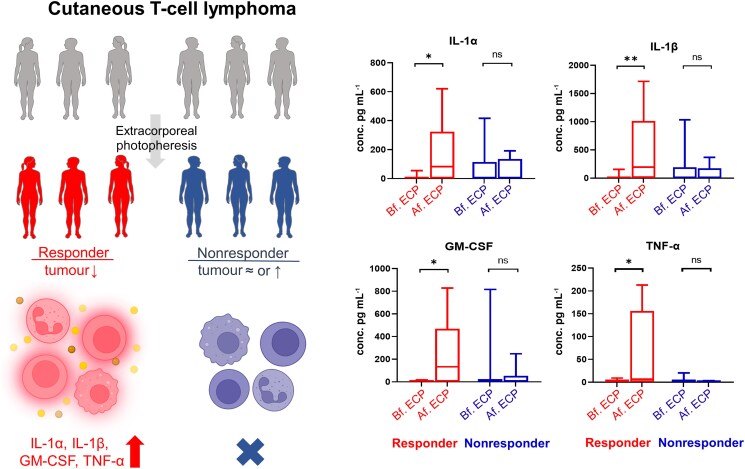

Linked Article: Pileri *et al. Br J Dermatol* 2023; **189**:504–505.

Plain language summary available online

Author Video: https://youtu.be/-uHm2q-MH9A

**What is already known about this topic?**

- Extracorporeal photopheresis (ECP) is a systemic first-line immunomodulatory therapy in leukaemic cutaneous T-cell lymphoma (L-CTCL) and in a variety of T-cell-mediated diseases.
- Enhancement of antibody-dependent cellular cytotoxicity is associated with treatment response to ECP.
- ECP’s exact mechanism of action is not sufficiently understood, and biomarkers for response are scarce.

**What does this study add?**

- We find a difference in the cytokine profile between patients with L-CTCL and healthy donors. In patients with L-CTCL, their serum interleukins (IL)-9 and IL-13 are increased, while tumour necrosis factor (TNF)-α is decreased.
- This study dissects the differences in the cytokine profiles of patients with L-CTCL who did and did not respond to ECP.
- We find significant differences in IL-1α, IL-1β, granulocyte–macrophage colony-stimulating factor (GM-CSF) and TNF-α in ECP responders vs. nonresponders.
- IL-1α, IL-1β, GM-CSF and TNF-α are promising biomarkers for monitoring ECP efficacy in patients with L-CTCL.

Leukaemic cutaneous T-cell lymphomas (L-CTCLs), including Sézary Syndrome (SS) and advanced-stage erythrodermic mycosis fungoides with blood involvement, are clinically characterized by erythroderma, lymphadenopathy and detection of abnormal circulating T cells in the blood. Although the pathogenesis of CTCL is not entirely understood, an impaired T-helper (Th) 1 immune response with exhaustion of effector cells is a hallmark of advanced disease. The accompanying immunodeficiency is caused by a decline of antigen-specific T-cell responses, impairment of natural killer (NK) cell activity and cell-mediated cytotoxicity, peripheral eosinophilia and elevated levels of serum IgE.^1–4^ Immunotherapies that revert an immunosuppressed phenotype to a normal phenotype profile play an important role in CTCL.^5^ Recently, numerous advances in the understanding of immune exhaustion in CTCL have been made. Specifically, CD4 tumour cells were shown to overexpress immune checkpoints such as programmed cell death protein 1 (PD-1),^2^ CTLA4,^4^ BTLA, FRCL3 and TIGIT,^3^ which may be relevant for future use of checkpoint inhibitors as treatment. However, it has been demonstrated recently that despite high levels of PD-1 on tumour cells, targeting this molecule may not be an optimal general strategy for all patients with CTCL, as the use of anti-PD-1 nivolumab increases proliferation of both nonmalignant and malignant clones of T cells.^2^ Therefore, perspectives of the use of checkpoint inhibitors in CTCL require further studies on both the molecular and clinical levels.

Currently, extracorporeal photopheresis (ECP), a traditional and well-established method of immunotherapy,^6–8^ is recommended as a first-line treatment of CTCL with blood involvement^9–11^ and further indicated for a variety of T-cell-mediated diseases, such as graft-versus-host disease (GvHD),^10,12,13^ systemic sclerosis^10,14^ and solid organ transplant rejection (SOTR).^10,15^ ECP is a method relying on collection of a patient’s lymphomonocytes into an extracorporeal circulating system, where they are subsequently treated with 8-methoxypsoralen (8-MOP) followed by ultraviolet A (UVA) light exposure. Although ECP has been used successfully in the clinic for nearly 30 years, its exact mechanisms of action are not sufficiently understood. On the one hand, there is experimental evidence for a direct reduction of T-cell proliferation by ECP-treated monocytes.^16^ Further, ECP positively affects NK cells, which have quantitatively and qualitatively impaired function in CTCL.^1^ Recently, we demonstrated that ECP activated NK cells and their antibody-dependent cellular cytotoxicity can serve as a biomarker for treatment response to ECP in patients with SS.^1^ ECP treatment also promotes the function of regulatory T (Treg) cells and myeloid-derived suppressor cells to suppress T-cell proliferation.^17,18^

On the other hand, ECP induces apoptosis in dendritic cells and the neutrophil population, which increases peripheral tolerance.^18,19^ It has been suggested that ECP may lead to changes in the repertoire of the secreted cytokines.^20–22^ Several studies have demonstrated that abnormalities in cytokine secretion is a hallmark of L-CTCL and might play a crucial role in the immune alterations observed in progressive disease. *Ex vivo* studies demonstrated that the T lymphocytes purified from patients with SS released significantly higher concentrations of interleukin (IL)-4, and significantly lower levels of IL-2 and interferon (IFN)-γ.^23,24^ Additional *in vitro* experiments have shown that CD4^+^ T cells from individual patients with CTCL can adopt a Treg phenotype, secreting IL-10 and transforming growth factor (TGF)-β.^25,26^ Transcriptional profiling for ECP responders in patients with L-CTCL revealed IL-1β and integrin signalling as a top hit pathway.^27^ Similarly, peripheral blood mononuclear cells (PBMCs) and monocytes collected from patients with GvHD before, and 30 min after, completion of ECP treatment reported a significant release of IL-1β and IL-12.^28–30^ Further, *in vitro* exposure of human PBMCs or monocytes to 8-MOP/UVA and subsequent lipopolysaccharide (LPS) or T-cell stimulation resulted in a significantly higher amount of IL-1β, IL-12 and tumour necrosis factor (TNF)-α and concomitant reduction of IL-6 and IL-10.^29,30^

Despite several reports on the immunoregulatory effects of ECP in patients with L-CTCL, to our knowledge, no complex multiparameter analysis of the cytokine secretion profile pre- and post-therapy has been published to date. In this study, we evaluated the secretion profile of 22 cytokines pre- and post-ECP therapy, in both patient serum and lymphocyte culture supernatant. We further investigated the relationship between ECP treatment response and the change in cytokine level with ECP treatment. Our findings contribute to the understanding of immunoregulatory mechanisms of ECP and may be used to objectively assess and monitor ECP response to treatment.

## Materials and methods

### Study participants

This study retrospectively and prospectively enrolled patients who underwent ECP for L-CTCL between 1997 and 2018. The frozen sera and PBMCs were obtained from the biobank of the University Hospital of Zürich. The study population included 25 patients who received ECP for L-CTCL as a first-line treatment (summarized in Table 1). Healthy blood controls (HC) were ordered from the blood donation centre in Zürich. Samples were not complete for all time points for all patients, resulting in a total number of 30 serum samples (15 for L-CTCL baseline and 15 for HC); and 32 PBMC culture supernatant samples (16 for L-CTCL baseline and 16 for L-CTCL after ECP treatment). The diagnosis of L-CTCL and the treatment response were evaluated according to the 2007 International Society for Cutaneous Lymphomas/European Organization for Research and Treatment of Cancer diagnostic criteria for the CTCL staging.^31^

**Table 1:** Characteristics of study

Patient characteristics	L-CTCL, *n* = 25
Sex, female : male, *n* (%)	14 : 11 (56 : 44)
Race, White : Black, *n* (%)	24 : 1 (96 : 4)
Alive : Dead : Lost to follow-up, *n* (%)	10 : 12 : 3 (40 : 48 : 12)
Age at diagnosis (years), mean (SD)	70.9 (10.1)
Disease stage at diagnosis	
IIIA, *n* (%)	1 (4)
IIIB, *n* (%)	4 (16)
IVA1, *n* (%)	15 (60)
IVA2, *n* (%)	3 (12)
IVB, *n* (%)	2 (8)
Time to diagnosis (years), mean (SD)^a^	4.7 (5.2)
Survival after diagnosis (years), mean (SD, range)^b^	3.9 (2.9, 0.5–11.0)
Detectable CD4^+^ Vβ TCR clone, *n* (%)^c^	13 (52)
Age at the start of ECP therapy (years), mean (SD)	72.4 (10.7)
Time from diagnosis to the start of ECP therapy, years, mean (SD)^d^	1.4 (2.3)
Number of ECP cycles received per patient, mean (SD, range)^e^	24.9 (17.0, 2–65)
Systemic treatment modalities^f^	
*ECP monotherapy, n (%)*	13 (52)
*ECP plus associated treatments, n (%)*	12 (48)
Interferon α, *n* (%)	10 (83)
Bexarotene, *n* (%)	5 (42)
Methotrexate, *n* (%)	4 (33)
Interferon γ, *n* (%)	2 (17)
ECP therapy follow-up	
*Ongoing treatment at the time of manuscript preparation, n (%)*	7 (28)
*Reason for ECP discontinuation, n (%)*	18 (72)
Death, *n* (%)	6 (33)
Disease progression, *n* (%)	4 (22)
Complete remission, *n* (%)	1 (6)
Other reasons, *n* (%)^g^	7 (39)
Response to ECP^h^	
*Responders, n (%)*	14 (56)
Complete response, *n* (%)	3 (12)
Partial response, *n* (%)	5 (20)
Minor response, *n* (%)	6 (24)
*Nonresponders, n (%)*	11 (44)
Stable disease, *n* (%)	6 (24)
Progressive disease, *n* (%)	5 (20)
Cause of death, *n* (%)	12 (48)
L-CTCL, *n* (%)	10 (83.3)
Other, *n* (%)^i^	2 (16.7)

ECP, extracorporeal photopheresis; L-CTCL, leukaemic cutaneous T-cell lymphoma; TCR, T-cell receptor.

^a^Defined as the time of the onset of any persistent cutaneous symptoms compatible with L-CTCL to the time of L-CTCL diagnosis. ^b^Defined as the time from diagnosis of L-CTCL to either death, lost to follow-up or to discontinued observation at the time of manuscript preparation. ^c^Assessed by flow cytometry during routine clinical investigations. ^d^Defined as the time from diagnosis of L-CTCL to the first cycle of ECP treatment. ^e^Summarized for individual patients either until cessation of ECP therapy or at the end of the manuscript preparation. ^f^Assessed from the beginning of ECP treatment until the last analysed blood sample for each patient. ^g^Comorbidity (*n* = 1), inclusion in clinical trials (*n* = 2), patients’ wish (*n* = 2) or lost to follow-up (*n* = 2). ^h^Percentages in this section are of the whole cohort. ^i^Colon carcinoma, suicide.

We evaluated the cytokine level in the patients’ sera prior to ECP treatment, and after 27 consecutive weeks of uninterrupted ECP therapy. Written informed consent was obtained from all participants. The study was conducted in accordance with the principles of the Declaration of Helsinki and was approved by the Institutional Review Board of the Swiss cantonal ethics committees (KEK No. 2018-00209).

### Extracorporeal photopheresis treatment and response criteria

ECP therapy was administered using the THERAKOS^®^ CELLEX^®^ Photopheresis System apparatus (Bedminster, NJ, USA), according to the guidelines on the use of ECP in the management of CTCL.^32,33^ At baseline, ECP was performed on 2 consecutive days, every 2 weeks; the regimen was modified for each individual patient according to treatment response. In case of insufficient response to ECP monotherapy, patients received additional systemic immunomodulating agents, mostly IFN-α and bexarotene. Patient response to ECP therapy was classified according to the improving level of blood involvement.^31^ Patients with complete response (CR), partial response (PR) and minor response (MR) were considered as responders. CR was defined as laboratory results returning to the range of the healthy population. Patients with PR showed greater than 50% quantitative reduction of tumour burden in the blood, and patients with MR showed 25–50% of improvement. Stable disease means the blood involvement after ECP improved less than 25% and the increase in neoplastic cell count is not higher than 50% from baseline. If the neoplastic cell count increased more than 25% compared with baseline, we defined it as progressive disease, i.e. a deterioration of the disease stage compared with baseline. Those with stable or progressive disease were considered to be nonresponders.

### Stimulation of peripheral blood mononuclear cells for cytokine production

PBMCs were isolated via Ficoll centrifugation and stored in liquid nitrogen. Upon thawing, fully automated cell counting was performed using Moxi Flow Smart Flow Cytometer Kit (ORFLO^®^ Technologies, Ketchum, ID, USA), and viability was evaluated with propidium iodide. In the case of cell viability < 70%, dead cells were removed via centrifugation on fetal bovine serum.^34^ After counting, PBMCs were subsequently stimulated with human T-cell Activation/Expansion kit (Miltenyi Biotec, Solothurn, Switzerland) at a final concentration of 1 × 10^6^ live cells per mL and 1 μL of LPS [from *Salmonella minnesota* R595 (Re), TLRgrade^TM^, Enzo Life Sciences AG, Lausen, Switzerland] in order to stimulate dendritic cells. Cells were cultured for 72 h and centrifuged, and supernatants from the cultures were harvested and stored at –20 °C.

### Cytokine assays and theoretical sensitivity

We quantified the concentrations of 22 cytokines, including IL-1α, IL-1β, IL-2, IL-4, IL-5, IL-6, IL-7, IL-8, IL-9, IL-10, IL-12, IL-13, IL-17a, IL-17f, IL-21, IL-22, IL-23, IL-31, granulocyte–macrophage colony-stimulating factor (GM-CSF), IFN-α, IFN-γ and TNF-α in serum and PBMC culture supernatant samples using multiplex bead-based immunoassays. LEGENDplex^TM^ Human Th Cytokine Mix and Match Subpanel kit (BioLegend, Amsterdam, the Netherlands), the BD^TM^ CBA Human Soluble Protein Flex Set System (BD Biosciences, Allschwil, Switzerland) and the Human HS Magnetic Luminex Performance Assay multiplex kit (R&D Systems, Zug, Switzerland) were used according to the manufacturers’ instructions. Because each cytokine assay has a sensitivity limitation, values lower than the detection range were noted as 0 pg mL^–1^, and values beyond the maximum detection range were labelled as the maximum detection concentration for the following analysis.

### Flow cytometry

Flow cytometry data was acquired on BD LSRFortessa^TM^ Cell Analyzer (BD Biosciences). The following antihuman monoclonal antibodies were used for flow cytometry experiments: CD3 (clone BW264/56, label PerCP; Miltenyi Biotec #130-096-910), CD4 (clone VIT4; label APC-Vio770; Miltenyi Biotec #130-098-153), CD7 (clone CD7-6B7; label PE-Cy7; BioLegend #343114), CD8 (clone REA734; label FITC; Miltenyi Biotec #130-110-815), CD26 (clone BA5b; label PE-Cy5; BioLegend #302708). Vβ clonal T-cell populations were assessed using IOTest^®^ Beta Mark TCR V beta Repertoire kit (Beckman Coulter, Nyon, Switzerland) according to the manufacturer’s instructions. Data analysis was performed on FCS Express 5 Flow Cytometry RUO (v5; https://denovosoftware.com) and plotting was done using GraphPad Prism 8 software.

### Statistics

Statistical analysis was performed using GraphPad Prism 8 software. For comparison between two groups, a two-tailed Mann–Whitney *U*-test was applied. *P*-values lower than 0.05 were considered statistically significant.

### Correlogram

Correlation matrix stands for the dependence and association between multiple variables. We used R package Hmisc (v4.7-1) to compute correlation coefficients of two variables and the corresponding significant value. The visualization of the correlation matrix was generated by R function corrplot() (v.0.92).^35^ The colour depth and size variation of circles indicate the correlation strength. Numbers ranging from –1 to 1 represent the Spearman’s rank correlation coefficients of the variables.

## Results

### Leukaemic cutaneous T-cell lymphomas are characterized by an altered serum cytokine profile compared with healthy individuals

**Figure 1:**
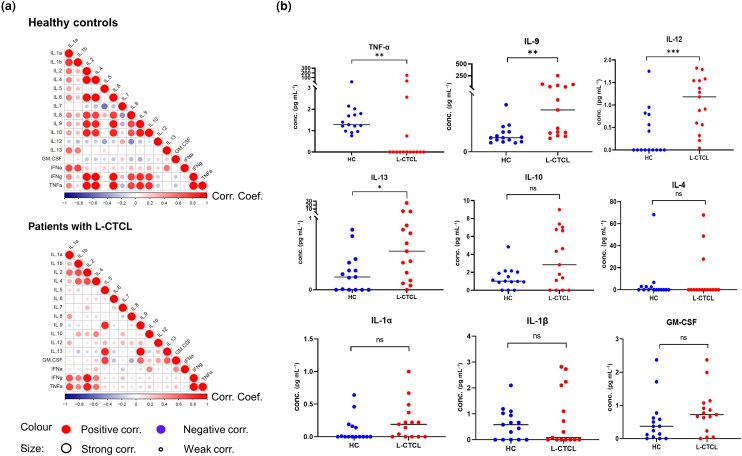
L-CTCL is characterized by an altered serum cytokine profile including loss of cytokine TNF-α and increase of cytokines IL-9, IL-12 and IL-13. (a) Patients with L-CTCL show distinct cytokine patterns compared with HC. The correlation matrix displays the positive and negative correlations between cytokine levels in the serum of HC (*n* = 15) and patients with L-CTCL (*n* = 16). Colour depth and size variation of circles in the figure indicate the correlation strength. Numbers ranging from –1 to 1 represent the Spearman’s rank correlation coefficients of the variables. (b) The dot plot revealed the absolute concentration of different cytokines in HC and in patients with L-CTCL. The sera collected from patients with L-CTCL showed a significant decrease of TNF-α and a significant increase of IL-9, IL-12 and IL-13 compared with HC. *P < 0.05, **P < 0.01 and ***P < 0.001. conc., concentration; GM-CSF, granulocyte–macrophage colony-stimulating factor; HC, healthy blood controls; IFN, interferon; IL, interleukin; L-CTCL, leukaemic cutaneous T-cell lymphoma; ns, not significant; TNF, tumour necrosis factor.

CTCL has a highly complex pathophysiology and has been mostly associated with increased Th2 cell activity.^36,37^ We expected the immune landscape in CTCL to be different from healthy individuals, both in terms of innate and adaptive cytokines. A combination of multiplex bead-based immunoassays was applied to simultaneously quantify the concentrations of 22 cytokines in the sera of patients with L-CTCL and healthy individuals. Indeed, the comprehensive analysis and the relevant correlation matrix profiling revealed a distinct cytokine pattern between L-CTCL and HC (Figure 1a). Interestingly, positive correlations between Th2 cytokines IL-5 and IL-9, IL-13 and GM-CSF are observed only in L-CTCL. Further examination of the concentration of single cytokines revealed a significant loss of TNF-α, and significant increase of IL-9, IL-12 and IL-13 in the sera of patients with L-CTCL compared with HC (Figure 1b). Other than those, there were no statistically significant differences in the levels of IL-1α, IL-1β, IL-2, IL-4, IL-5, IL-6, IL-7, IL-8, IL-10, IFN-α and IFN-γ. In most cases, the concentrations of IL-17a, IL-17f, IL-21, IL-22, IL-23 and IL-31 are lower than the detection limit (Figure S1; see Supporting Information).

### Extracorporeal photopheresis boosts the release of innate immunity cytokines in responders’ peripheral blood mononuclear cells

**Figure 2:**
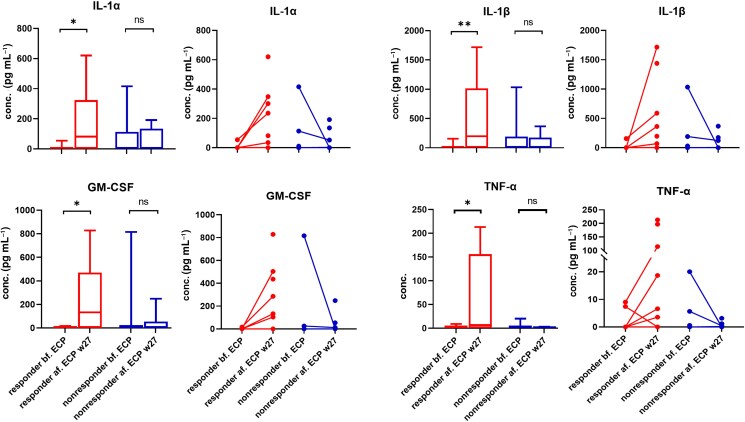
Box plots and dot plots of paired samples from responders and nonresponders to ECP at baseline and at 27 weeks post-treatment. ECP boosts the release of innate immunity cytokines in the PBMCs of ECP responders. Post-ECP PBMCs acquired from patients responding to ECP (*n* = 9) released statistically higher amounts of innate immune cytokines such as IL-1α, IL-1β, GM-CSF and TNF-α in comparison with the baseline (*n* = 9). In contrast, the pre- (*n* = 7) and post- (*n* = 7) ECP PBMCs purified from ECP nonresponders showed no significant differences in cytokine production. The dot plots of the paired samples show a boost of innate immune cytokine secretion after ECP in the individual responders, and also reveal a tendency of decrease in the release of cytokines in the post-ECP PBMCs of the nonresponders. *P < 0.05, **P < 0.01 and ***P < 0.001. ECP, extracorporeal photopheresis; GM-CSF, granulocyte–macrophage colony-stimulating factor; IL, interleukin; ns, not significant; PBMCs, peripheral blood mononuclear cells; TNF, tumour necrosis factor.

As a next step, we aimed to explore the dynamic changes in the cytokine profile in patients with L-CTCL undergoing immunomodulatory treatment with ECP. Individual patients with L-CTCL were classified as responders or nonresponders according to the quantitative assessment of malignant burden in the skin by the modified Severity Weighted Assessment Tool (mSWAT) and also the proportion of neoplastic cells in the blood. The cytokine levels of culture supernatants from the patients’ PBMCs were evaluated at baseline and after 27 weeks of uninterrupted ECP treatment. Strikingly, we found that the post-ECP PBMCs from responding patients released a significantly higher amount of innate immune cytokines including IL-1α, IL-1β, GM-CSF and TNF-α in comparison with the baseline. In contrast, the pre- and post-ECP PBMCs from nonresponders showed no significant differences in the relevant cytokine production. The dot plot of paired samples disclosed a boost of innate immune cytokine secretion after ECP in the responder group, and it also revealed the tendency to decrease the level of cytokine discharging in the post-ECP PBMCs of the nonresponders (Figure 2). The alterations of TNF-α, IL-1α, IL-1β and GM-CSF can possibly be used as biomarkers of response to ECP in patients with L-CTCL.

### Effective extracorporeal photopheresis leads to immune profile recovery in the skin and blood of individual patients with leukaemic cutaneous T-cell lymphoma

**Figure 3:**
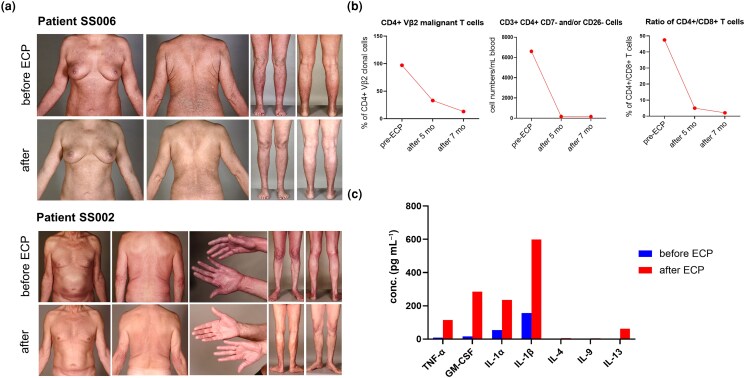
Effective ECP leads to immune profile recovery in the skin and blood of patients with L-CTCL. (a) Clinical images of patients treated successfully with ECP who showed a complete clearance of the erythema. (b) ECP effectiveness was observed from the blood of patient SS006 (with L-CTCL), which displayed a reduction of CD4 ^+^ Vβ2 clonal T cells and malignant CD3 ^+^ CD4 ^+^ CD7^–^ and/or CD26^–^ T cells, and the recovery of the CD4^+^/CD8^+^ T-cell ratio. (c) Cytokine profile from patient SS002 (representative patient with L-CTCL) revealed that ECP strongly boosts innate immunity cytokines TNF-α, GM-CSF, IL-1α and IL-1β. ECP, extracorporeal photopheresis; GM-CSF, granulocyte–macrophage colony-stimulating factor; IL, interleukin; L-CTCL, leukaemic cutaneous T-cell lymphomas; TNF, tumour necrosis factor.

Clinical observations of responders to immunomodulatory ECP treatment showed a complete clearance of erythema in patients’ skin (Figure 3a). We further confirmed laboratory disease remission in the patient’s blood by flow cytometry. In the clinical model of patient SS006 (Figure 3a), the malignant CD4 ^+^ Vβ2 clonal T cells occupied 97% of the CD4^+^ T-helper cell population before the therapy. After ECP treatment for 5 consecutive months, the level of malignant T cells was reduced by 77% compared with baseline, and this value further dropped to only 13% in the whole CD4^+^ cell population following 2 additional months of continuous regimen. The loss of CD7 or CD26 on the T-cell surface is a widely accepted surrogate marker of malignant CTCL cells. The proportion of malignant CD3 ^+^ CD4 ^+^ CD7^–^ and/or CD26^–^ T-helper cells in the responder’s blood decreased from 6605 cells per mL blood to 162 cells per mL blood after 5 months of ECP treatment, and the effectiveness was maintained in a stable scenario when continuing ECP for 7 months. The percentage of CD4^+^ T-helper cells vs. CD8^+^ cytotoxic T cells also declined from 47.5% to 5% at 5 months and to 2% at 7 months after ECP, which indicates that the patient’s immunity shifted towards a normal condition of a healthy individual (Figure 3b). Furthermore, we demonstrated cytokine alteration of an ECP responder (SS002 from Figure 3a). The result manifested a potent increase of relevant innate immune cytokines such as TNF-α, IL-1α, IL-1β and GM-CSF in this case model (Figure 3c). To conclude, our results demonstrate that ECP stimulates the innate immune network and facilitates the redirection of the tumour-biased immunosuppressive microenvironment towards active antitumour responses.

## Discussion

In this study, we evaluated the immune-regulatory effect of ECP through the cytokine secretion pattern on comparatively large cohorts. Firstly, we compared the cytokine repertoire of L-CTCL and healthy individuals. The results of our study showed significantly increased levels of immunosuppressive cytokines IL-9, IL-12 and IL-13 with a concomitant decrease in the secretion of pro-inflammatory TNF-α in patients with L-CTCL compared with healthy individuals. The elevation of IL-9 and IL-13 is consistent with the widely accepted assumption of a Th2-biased immunosuppression microenvironment in patients with advanced CTCL.^24,36^

Interestingly, in contrast to the findings of other groups, we detected a significant increase of IL-12 in the serum of patients with L-CTCL, and we did not observe a significant difference in the level of immunosuppressive cytokine IL-4^36,38^ and Treg cytokine IL-10.^25,26^ One possible explanation for this discrepancy could be that the previous publications were based mainly on *ex vitro* experiments. Patients’ PBMCs or T lymphocytes were cultured in medium with phytohaemagglutinin stimulation, followed by cytokine measurement in the culture supernatant. Therefore, the results might not represent the substantial *in vivo* condition. In addition, depending on the naïve character of each cytokine, some of them exist at a relatively low level in both healthy and diseased samples (only a few picograms per mL) and may remain under the detectable limitation of the experimental method. According to our results, serum samples contain only minuscule amounts of IL-4, IL-17a, IL-17f, IL-21, IL-22 and IL-23. Thus, a more specific and sensitive detection method is required for the measurement of these cytokines. In addition, the results might be influenced by the relatively large sample size compared with other studies. In our observation, some L-CTCL cases show a rise in IL-10 concentration; however, it did not reach a significantly different level in our samples. Because CTCL is a highly heterogeneous disease, a specific patient cohort whose PBMCs secrete a higher amount of IL-10 might be present, strengthening the maturation of the Treg phenotype and skewing the microenvironment towards an immunocompromised status.

There have been only a limited number of studies showing changes in the cytokine profile in other diseases treated with ECP. ECP has most commonly been used for GvHD and SOTR. For GvHD, the cytokine profile shows progress towards a Th2 response, with an increase in IL-4, IL-10 and TGF-β; but a decrease in IL-1α, IL-1β, IL-12, IFN-γ and TNF-α.^39–41^ For SOTR, the cytokine profile shows a decrease in IL-6, IL-10, IL-17a and IFN-γ^39,42^ (summarized in Table 2). This contrasts with our findings in L-CTCL responders to ECP, and we believe that the difference is based on the fact that CTCL is an intrinsic malignancy of T cells, while GvHD and SOTR are reactive immune-mediated nonmalignant conditions.

**Table 2:** Cytokine profile alteration after ECP treatment

Factor	L-CTCL responders	GvHD	SOTR
IL-1α	Increase	Decrease	
IL-1β	Increase	Decrease	
IL-4	No difference	Increase	
IL-6	No difference	Decrease	Decrease
IL-10	No difference	Increase considered most important factor	Decrease
IL-12	No difference	Decrease	
IL-17a	No difference		Decrease
GM-CSF	Increase		
IFN-γ	No difference	Decrease	Decrease
TNF-α	Increase	Decrease	
TGF-β	Not tested	Increase	

ECP, extracorporeal photopheresis; GM-CSF, granulocyte–macrophage colony-stimulating factor; GvHD, graft-versus-host disease; IFN, interferon; IL, interleukin; L-CTCL, leukaemic cutaneous T-cell lymphoma; SOTR, solid organ transplant rejection; TGF, transforming growth factor; TNF, tumour necrosis factor.

To our knowledge, this is the first study demonstrating that the ECP responders’ PBMCs release a statistically higher amount of IL-1α, IL-1β, GM-CSF and TNF-α after long-term ECP therapy. In contrast, the ECP nonresponders’ PBMCs did not show any significant difference. The increase of IL-1β and TNF-α secretion in PBMCs and monocytes on ECP treatment has been observed already in patients with L-CTCL or GvHD and reported for both *in vivo* and *ex vivo* conditions.^27,29^ Our results strengthen this observation, link the immunomodulatory effect to the therapeutic success of the ECP therapy and provide evidence on its long-term efficacy, detected after an average of 6 months of uninterrupted ECP. Members of the IL-1 family were validated as potent proinflammatory molecules and strong mediators between innate and adaptive immunity.^43^ In a mouse model of melanoma, it has been proven that IL-1α and IL-1β are essential for tumour eradication and for activating tumour-specific T lymphocytes and tumour-infiltrating macrophages.^44^ GM-CSF is a major factor priming myelopoiesis and maturation of myeloid cells, and consequently contributes to T-cell activation and differentiation.^45,46^ Vowels *et al*. reported a post-ECP increase in proinflammatory TNF**-**α, and its tumoricidal effects were associated with a better outcome in patients with CTCL.^47^ Our data support that ECP primed the production of innate immune cytokines in the ECP responders’ lymphocytes, and this empowers both innate and adaptive immunity for eliminating tumour cells.

In conclusion, our data confirm previous observations suggesting that patients with CTCL are in an immunosuppressed state due to the increase of IL-9 and IL-13 and loss of TNF-α. Our analysis further highlights IL-1α, IL-1β, GM-CSF and TNF-α to be the top hit cytokines, secreted by ECP responders’ lymphocytes, which might allow their use as biomarkers for predicting ECP responses. It is also worth noting that all the above-mentioned changes in cytokine concentrations were apparent after 27 weeks of treatment. This supports the recommendation that ECP should be continued for a time period of not less than 6 months before concluding that it lacks efficacy.^6^

## Supplementary Material

ljad220_Supplementary_Data

## Data Availability

The data underlying this article will be shared on reasonable request to the corresponding author.
