# Supplementary material for: Boost of innate immunity cytokines as biomarkers of response to extracorporeal photopheresis in patients with leukaemic cutaneous T-cell lymphoma
Source: Br J Dermatol. 2023 Jul 6;189(5):603–11. doi: 10.1093/bjd/ljad220 (PMC13077219; doi:10.1093/bjd/ljad220)
Supplement: ljad220_Supplementary_Data [file ljad220_supplementary_data.zip › ljad220 JC.pptx]

## Slide 1
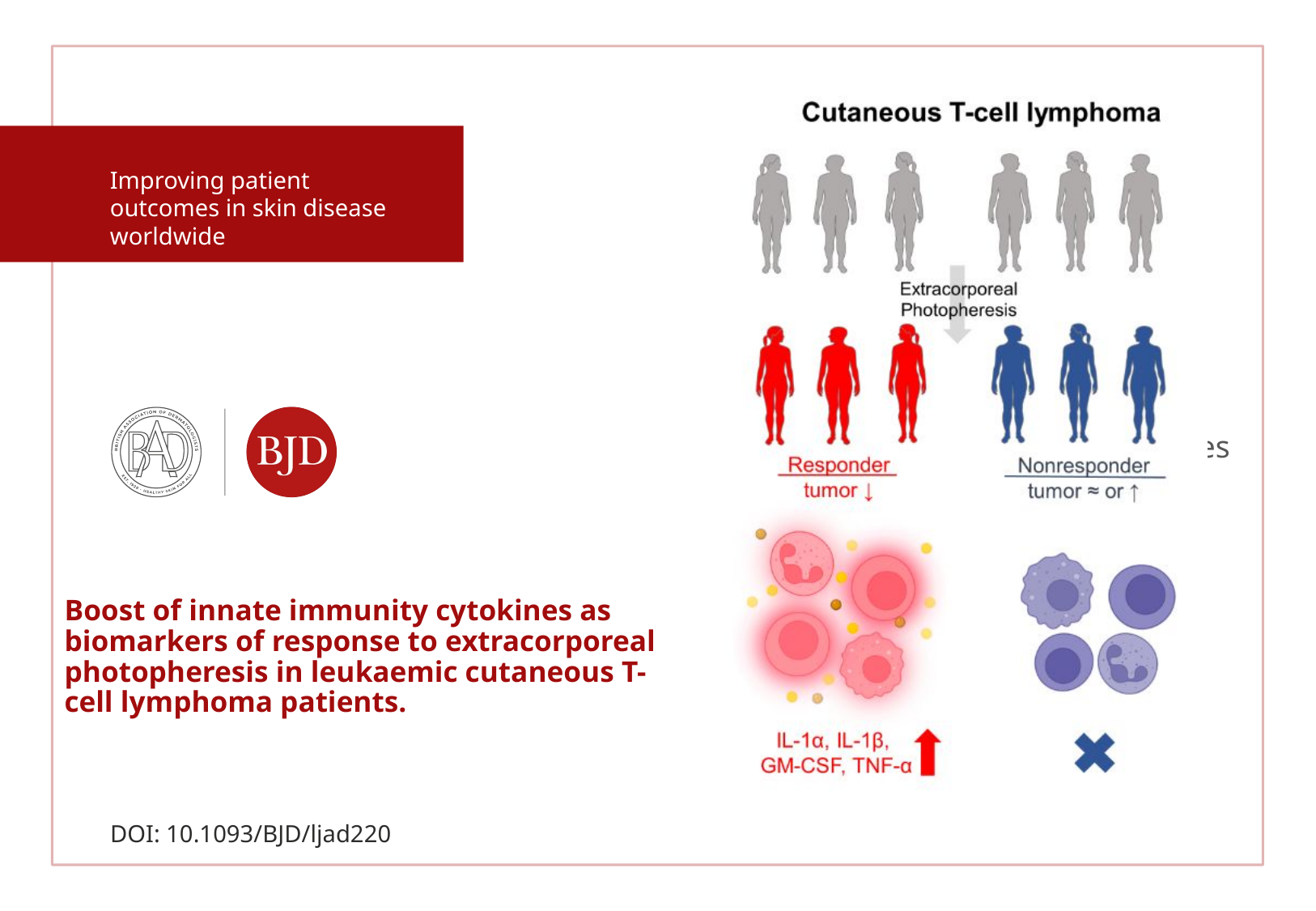

You can add a cropped version of one of your figures to this area
# Boost of innate immunity cytokines as biomarkers of response to extracorporeal photopheresis in leukaemic cutaneous T-cell lymphoma patients.
DOI: 10.1093/BJD/ljad220

## Slide 2
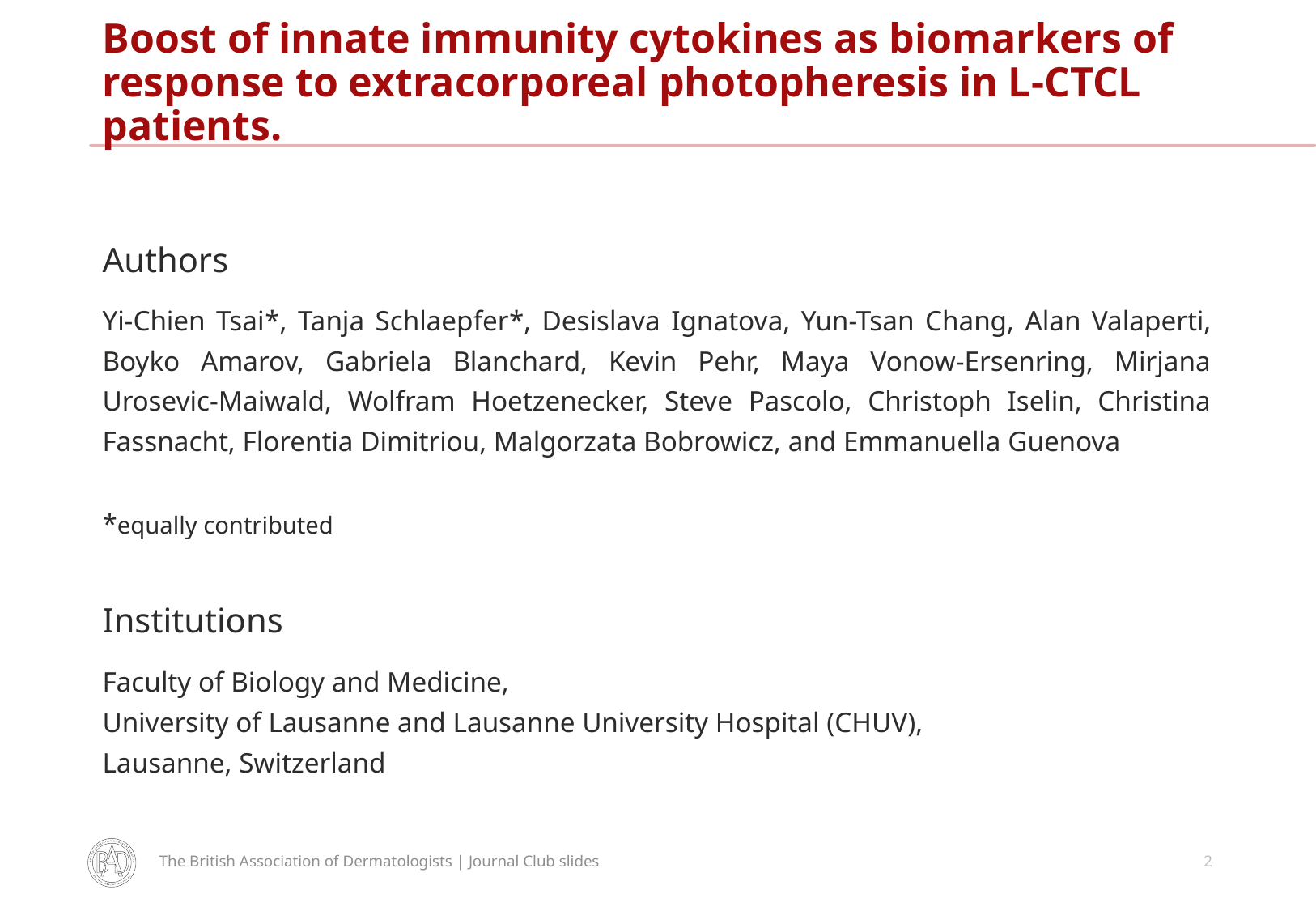

# Boost of innate immunity cytokines as biomarkers of response to extracorporeal photopheresis in L-CTCL patients.
Authors
Yi-Chien Tsai*, Tanja Schlaepfer*, Desislava Ignatova, Yun-Tsan Chang, Alan Valaperti, Boyko Amarov, Gabriela Blanchard, Kevin Pehr, Maya Vonow-Ersenring, Mirjana Urosevic-Maiwald, Wolfram Hoetzenecker, Steve Pascolo, Christoph Iselin, Christina Fassnacht, Florentia Dimitriou, Malgorzata Bobrowicz, and Emmanuella Guenova
*equally contributed
Institutions
Faculty of Biology and Medicine,
University of Lausanne and Lausanne University Hospital (CHUV),
Lausanne, Switzerland
The British Association of Dermatologists | Journal Club slides
2

## Slide 3
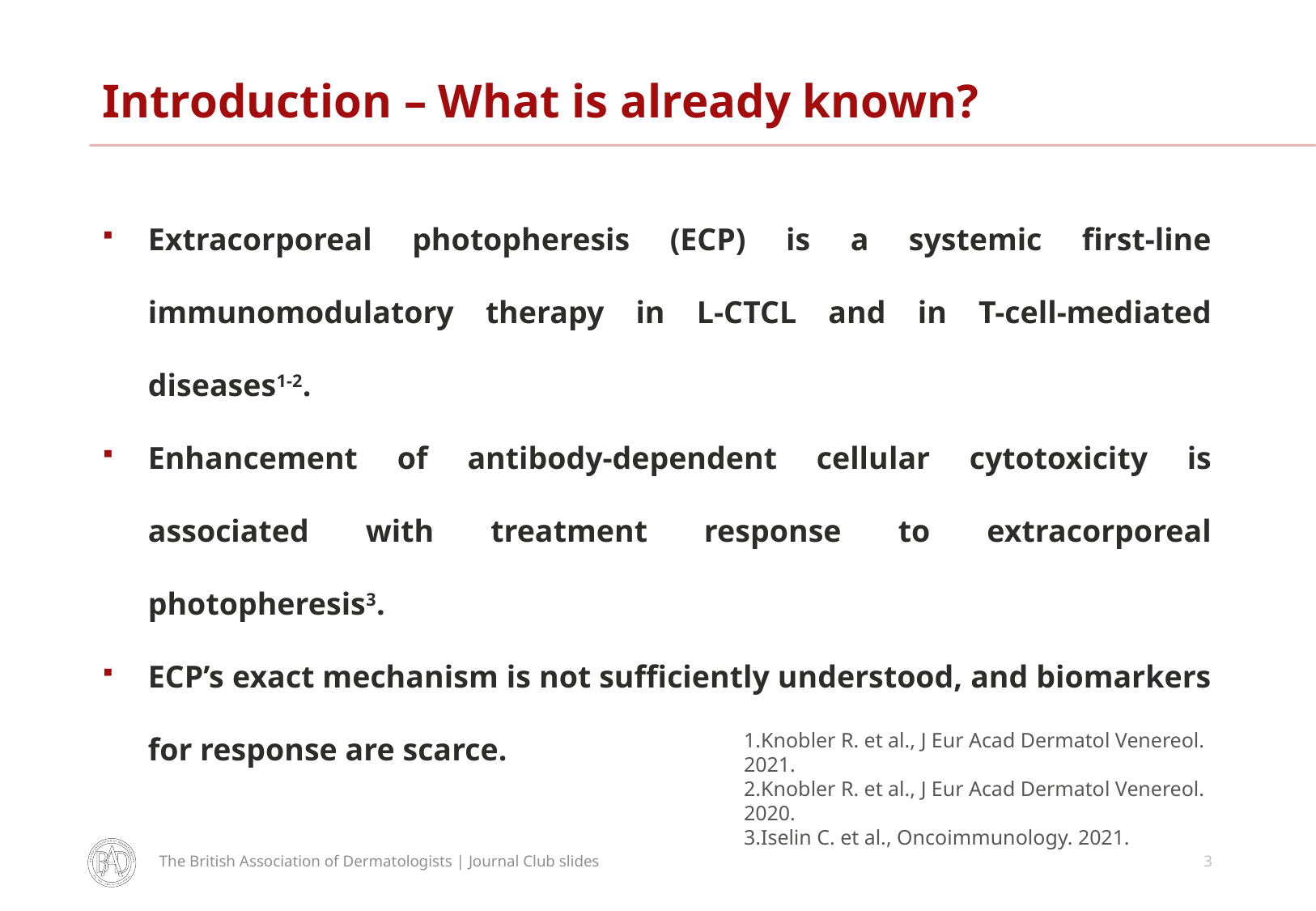

# Introduction – What is already known?
Extracorporeal photopheresis (ECP) is a systemic first-line immunomodulatory therapy in L-CTCL and in T-cell-mediated diseases1-2.
Enhancement of antibody-dependent cellular cytotoxicity is associated with treatment response to extracorporeal photopheresis3.
ECP’s exact mechanism is not sufficiently understood, and biomarkers for response are scarce.
1.Knobler R. et al., J Eur Acad Dermatol Venereol. 2021.
2.Knobler R. et al., J Eur Acad Dermatol Venereol. 2020.
3.Iselin C. et al., Oncoimmunology. 2021.
The British Association of Dermatologists | Journal Club slides
3

## Slide 4
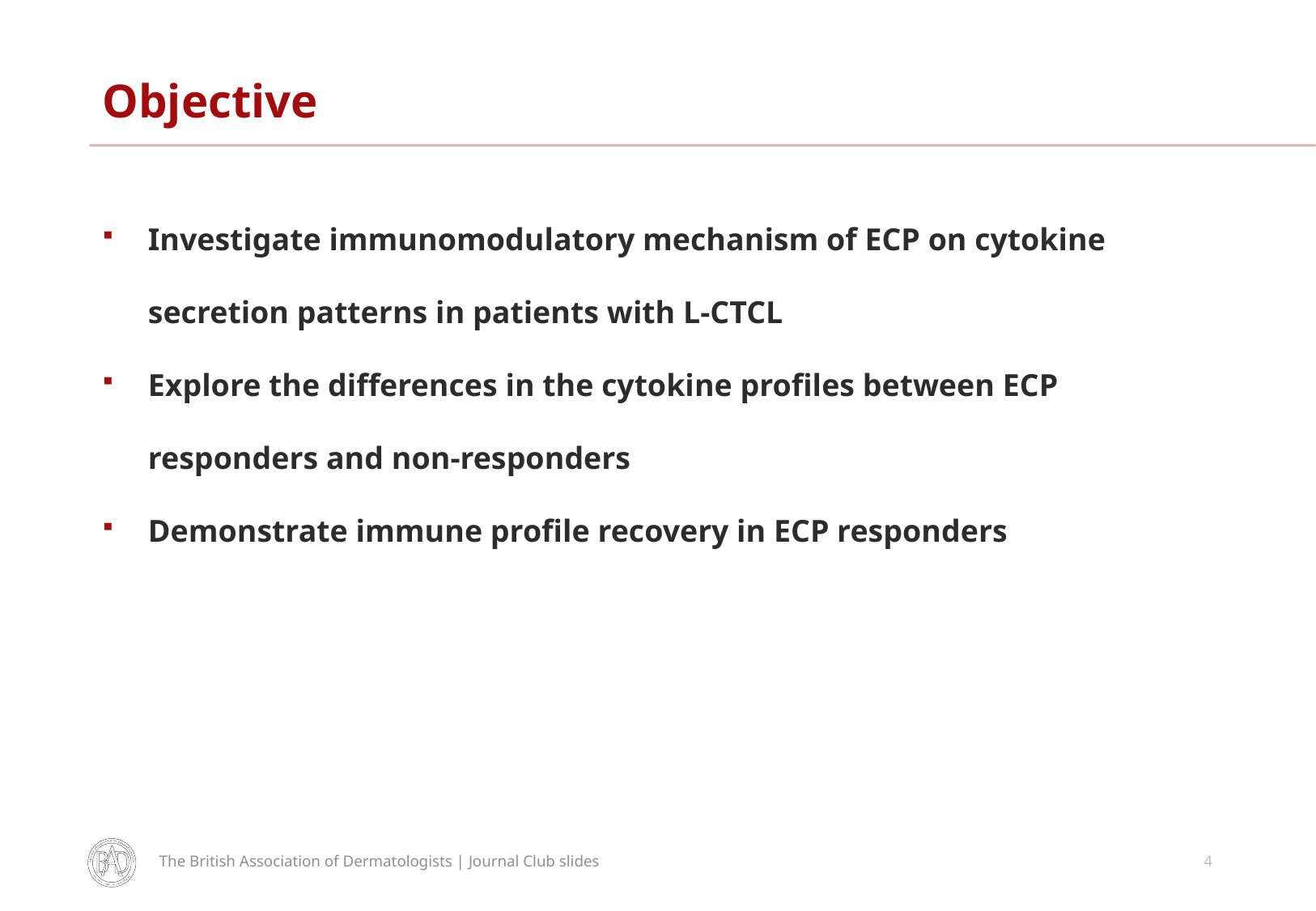

# Objective
Investigate immunomodulatory mechanism of ECP on cytokine secretion patterns in patients with L-CTCL
Explore the differences in the cytokine profiles between ECP responders and non-responders
Demonstrate immune profile recovery in ECP responders
The British Association of Dermatologists | Journal Club slides
4

## Slide 5
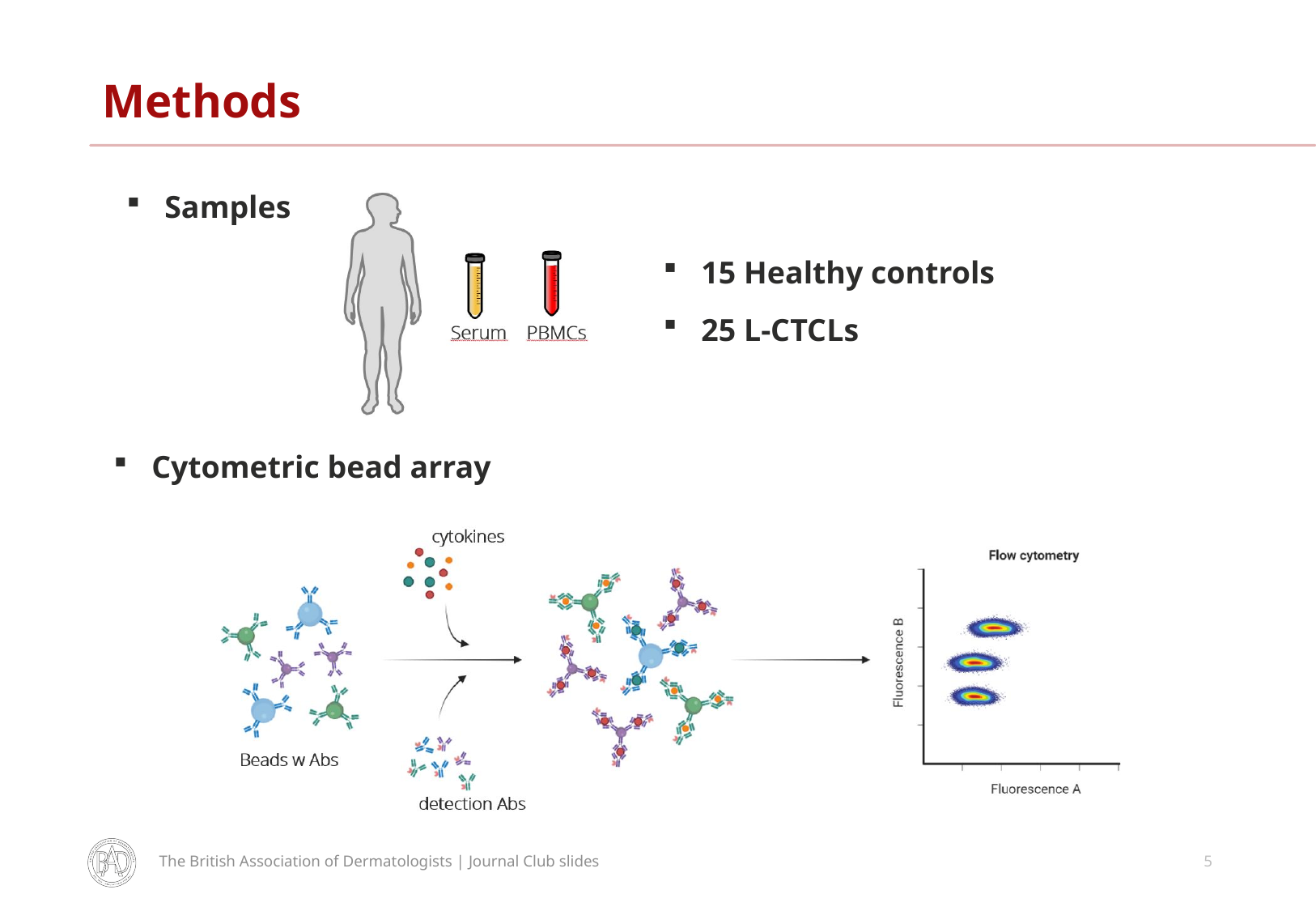

# Methods
Samples
15 Healthy controls
25 L-CTCLs
Cytometric bead array
The British Association of Dermatologists | Journal Club slides
5

## Slide 6
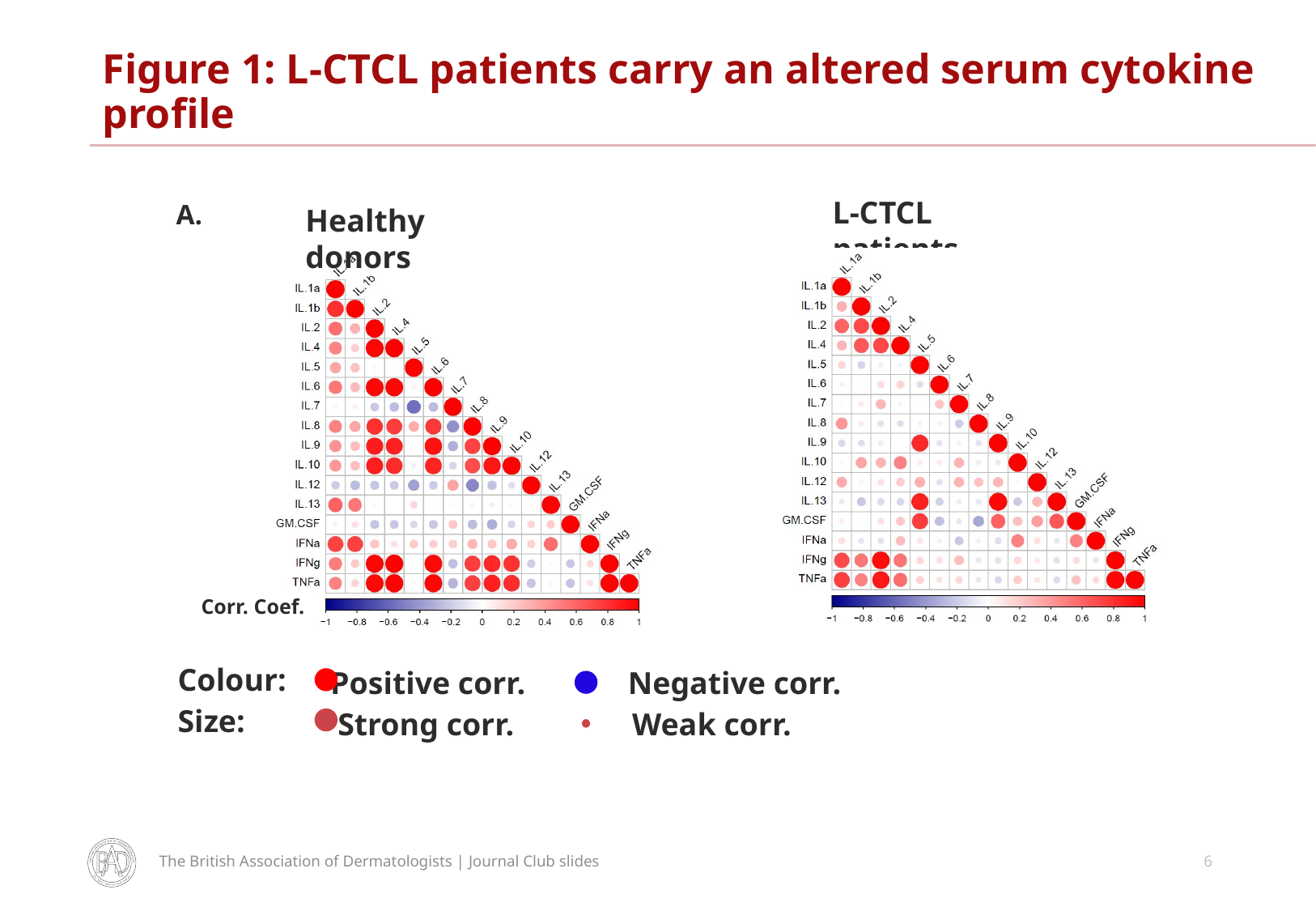

# Figure 1: L-CTCL patients carry an altered serum cytokine profile
L-CTCL patients
A.
Healthy donors
Corr. Coef.
Colour:
Size:
Positive corr. Negative corr.
Strong corr. Weak corr.
The British Association of Dermatologists | Journal Club slides
6

## Slide 7
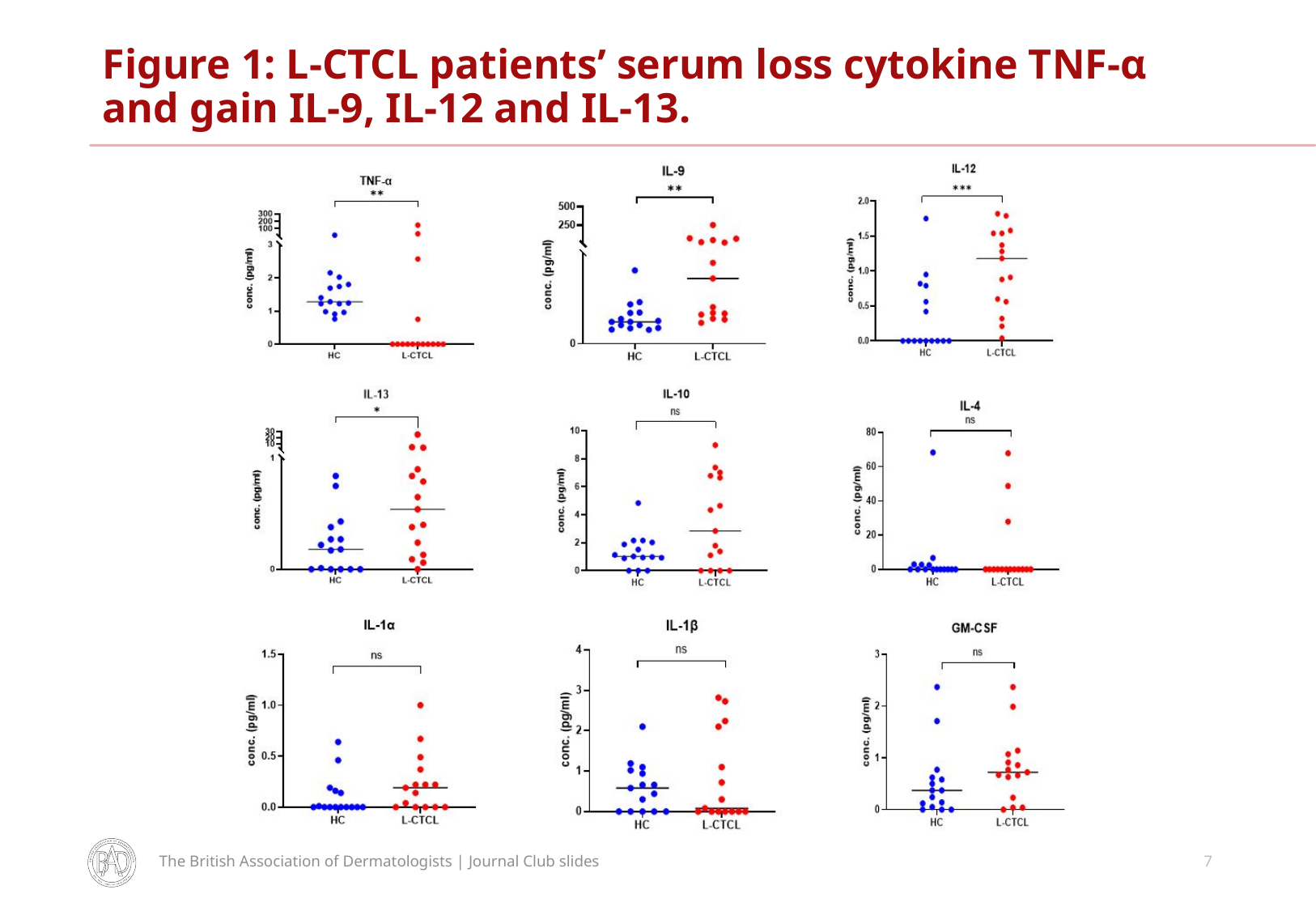

# Figure 1: L-CTCL patients’ serum loss cytokine TNF-α and gain IL-9, IL-12 and IL-13.
The British Association of Dermatologists | Journal Club slides
7

## Slide 8
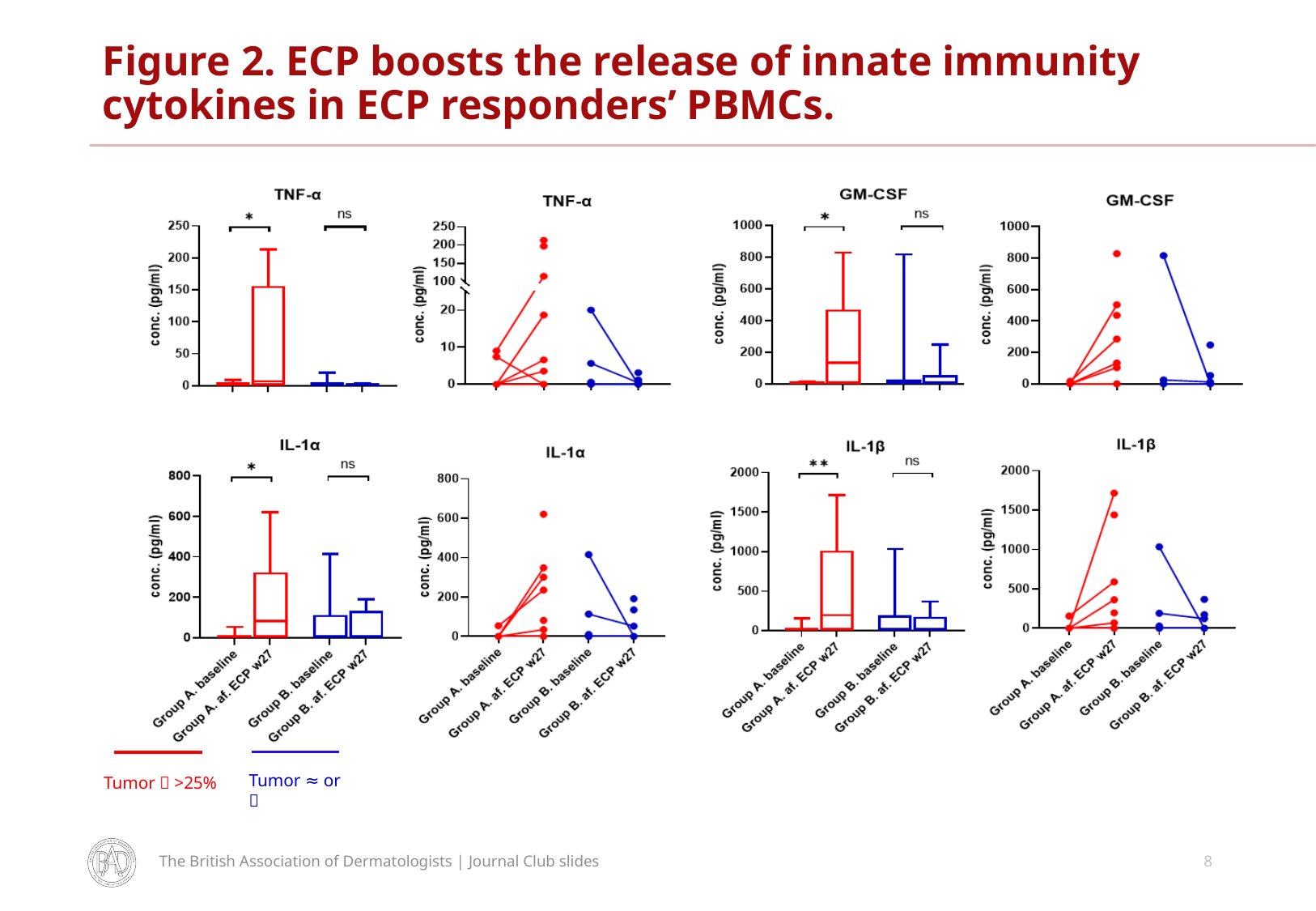

# Figure 2. ECP boosts the release of innate immunity cytokines in ECP responders’ PBMCs.
Tumor ≈ or 
Tumor  >25%
The British Association of Dermatologists | Journal Club slides
8

## Slide 9
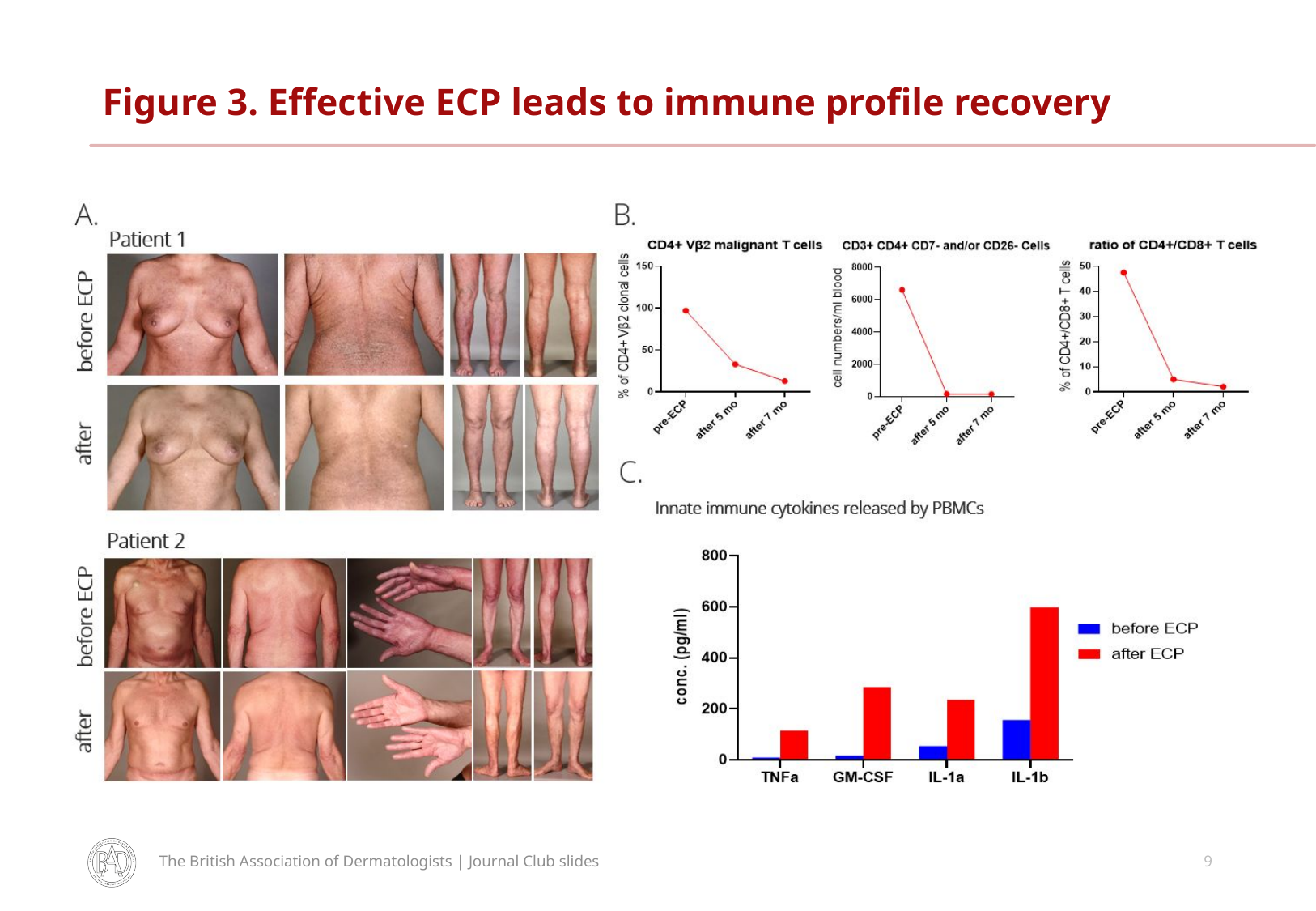

# Figure 3. Effective ECP leads to immune profile recovery
The British Association of Dermatologists | Journal Club slides
9

## Slide 10
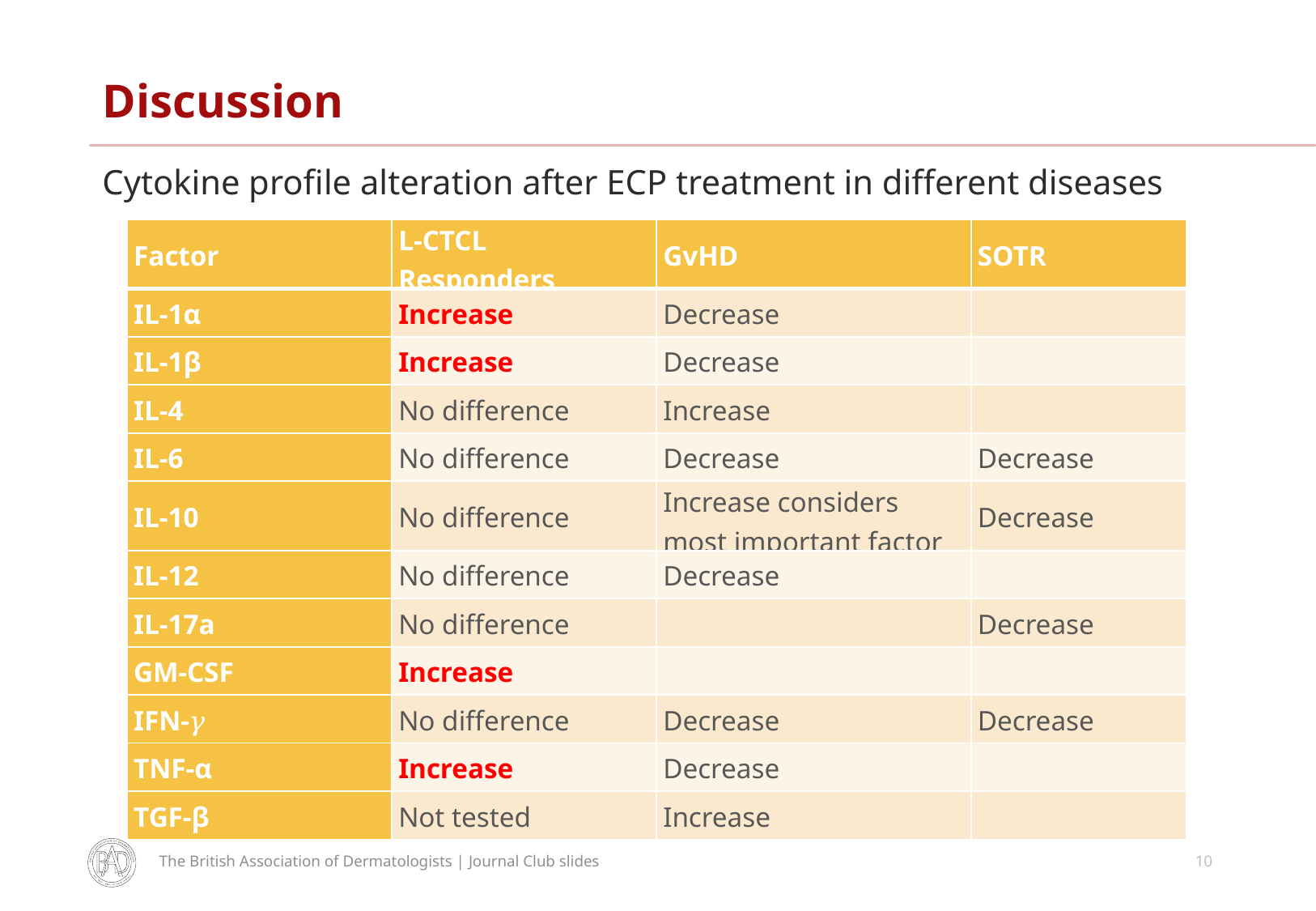

# Discussion
Cytokine profile alteration after ECP treatment in different diseases
| Factor | L-CTCL Responders | GvHD | SOTR |
| --- | --- | --- | --- |
| IL-1α | Increase | Decrease | |
| IL-1β | Increase | Decrease | |
| IL-4 | No difference | Increase | |
| IL-6 | No difference | Decrease | Decrease |
| IL-10 | No difference | Increase considers most important factor | Decrease |
| IL-12 | No difference | Decrease | |
| IL-17a | No difference | | Decrease |
| GM-CSF | Increase | | |
| IFN-𝛾 | No difference | Decrease | Decrease |
| TNF-α | Increase | Decrease | |
| TGF-β | Not tested | Increase | |
The British Association of Dermatologists | Journal Club slides
10

## Slide 11
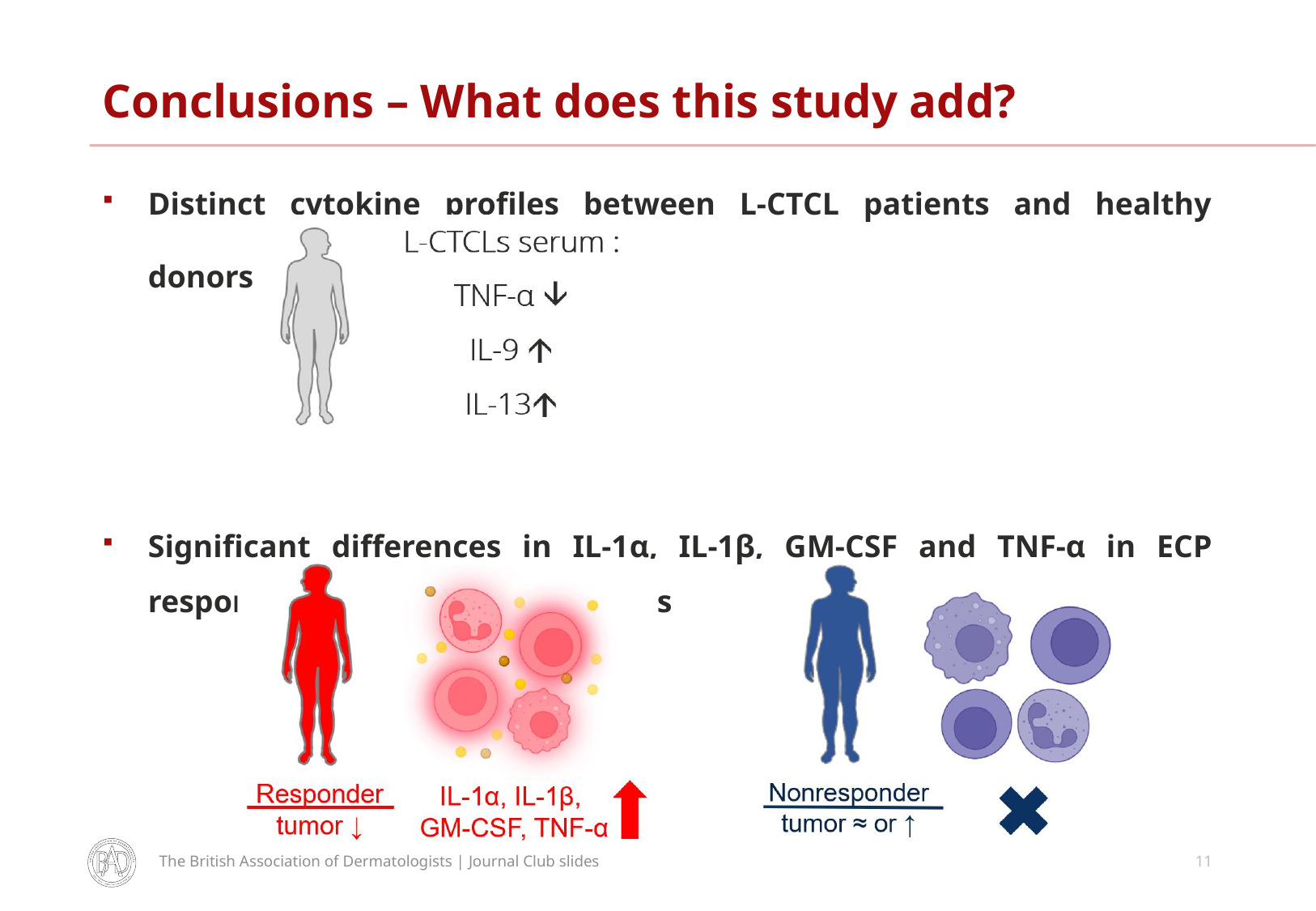

# Conclusions – What does this study add?
Distinct cytokine profiles between L-CTCL patients and healthy donors
Significant differences in IL-1α, IL-1β, GM-CSF and TNF-α in ECP responders versus non-responders
The British Association of Dermatologists | Journal Club slides
11

## Slide 12
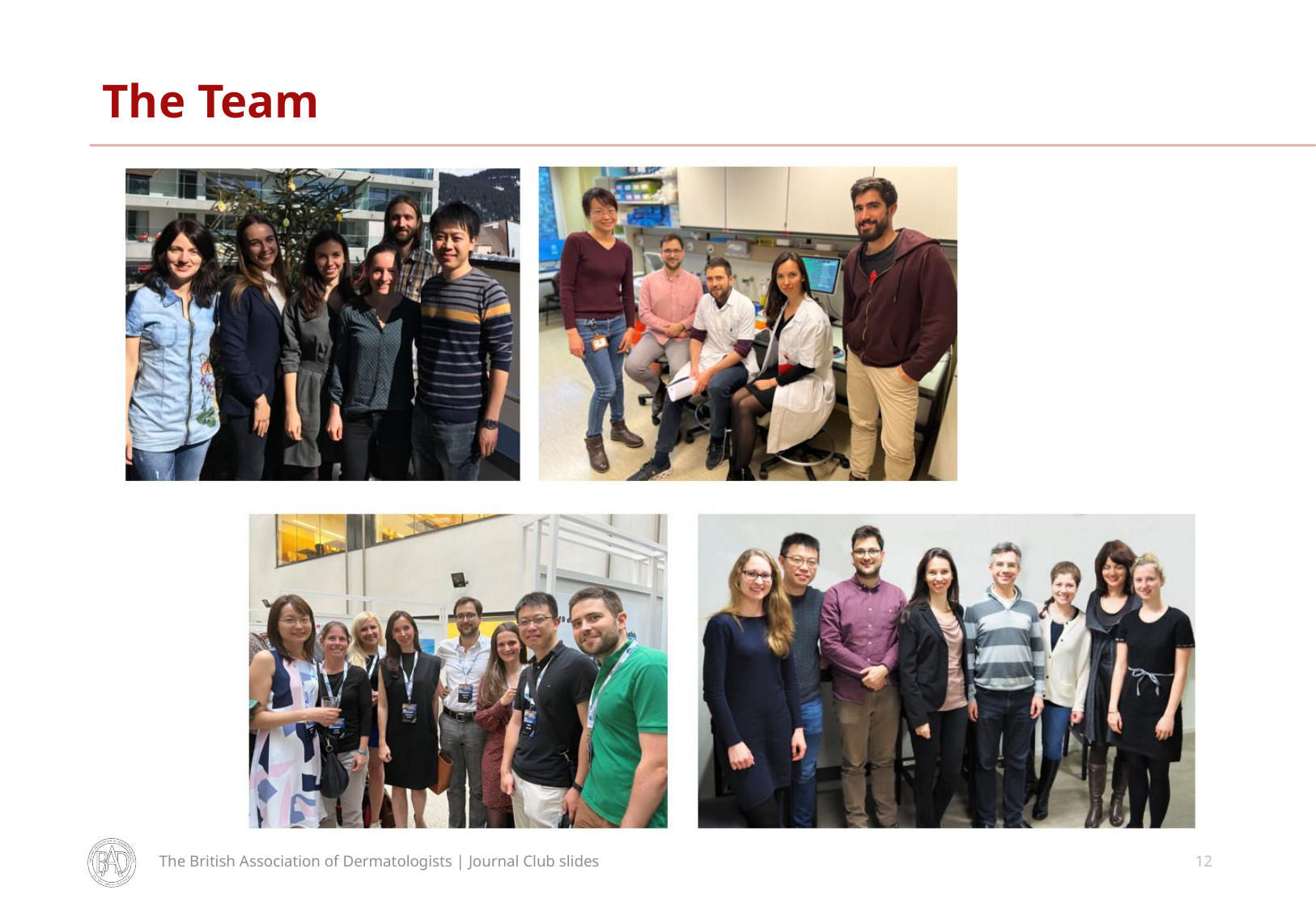

# The Team
The British Association of Dermatologists | Journal Club slides
12

## Slide 13
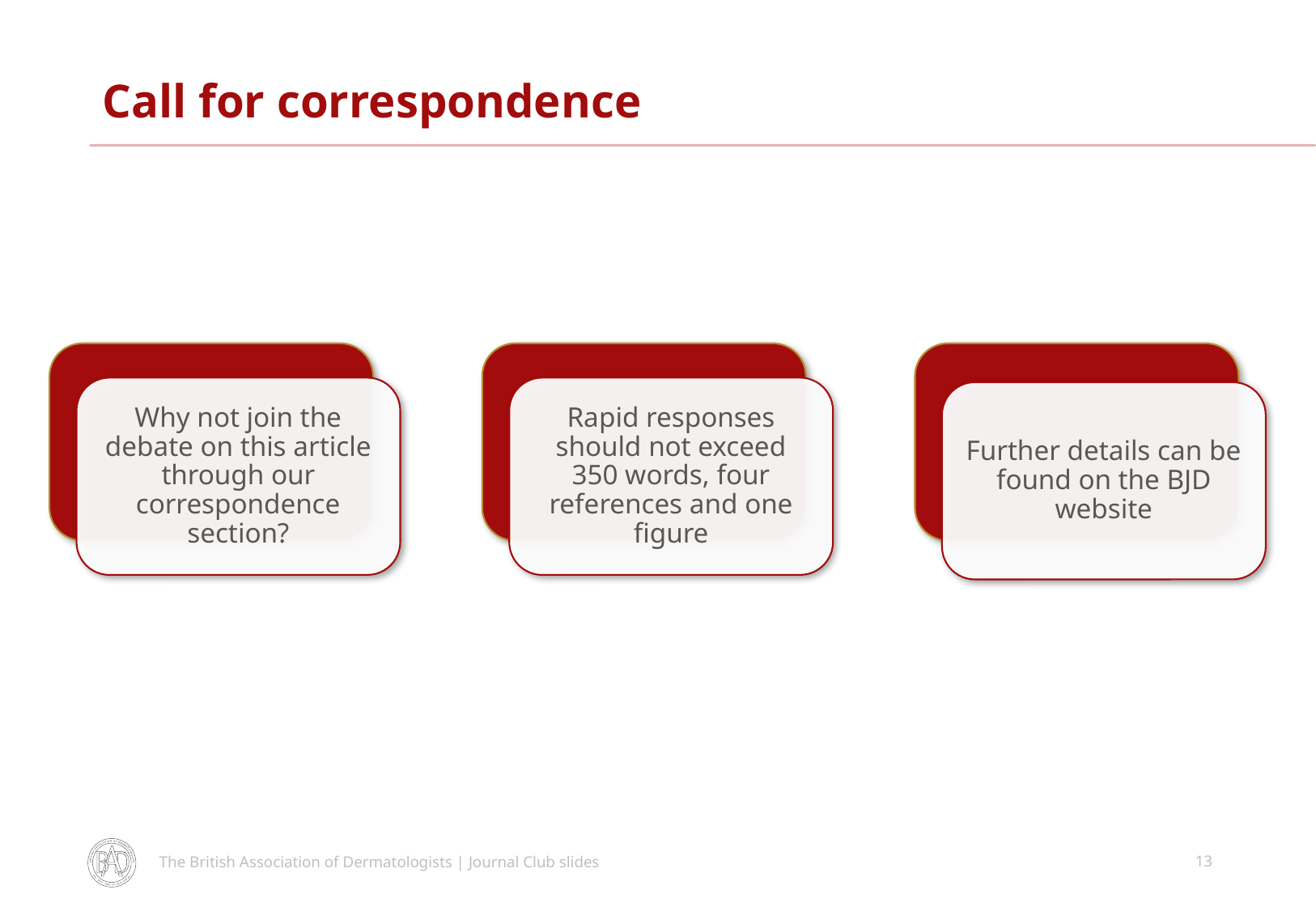

# Call for correspondence
Why not join the debate on this article through our correspondence section?
Rapid responses should not exceed 350 words, four references and one figure
Further details can be found on the BJD website
The British Association of Dermatologists | Journal Club slides
13
