# Supplementary material for: Boost of innate immunity cytokines as biomarkers of response to extracorporeal photopheresis in patients with leukaemic cutaneous T-cell lymphoma
Source: Br J Dermatol. 2023 Jul 6;189(5):603–11. doi: 10.1093/bjd/ljad220 (PMC13077219; doi:10.1093/bjd/ljad220)
Supplement: ljad220_Supplementary_Data [file ljad220_supplementary_data.zip › ljad220_Supplementary_Data.pdf]

Supplementary material

Materials and methods

Neuropsychological assessment

Processing speed was assessed using the Trail Making Test A (TMT-A) and Symbol-Digit Modalities Test (SDMT); executive function was assessed using the TMT-A, TMT-B, the Verbal Fluency test (animal and job naming); memory was assessed using the forward and backward scores of the Digit Span test, the immediate and delayed recall scores of the Rey Auditory Verbal Learning Test (RAVLT) and the delayed recall score of Rey-Osterrieth Complex Figure Test (RCFT). The raw test scores were standardized as z-scores. Next, compound scores were computed per cognitive domain: processing speed was calculated by the sum of (1) the inverse Z score of the TMT-A, (2) the Z score of SDMT; executive function was calculated by the sum of (1) the inverse Z score of the TMT ratio (TMT-B/TMT-A), (2) the average Z score of Verbal Fluency Test of animal naming and job naming; memory was calculated as the sum of (1) the Z score of DST, (2) the average Z score of RAVLT of immediate recall and delayed recall; (3) the average Z score of ROCF of immediate recall and delayed recall. (Supplementary Table e-1).

Table e-1. Calculation of each cognitive domain

Cognitive domain	Cognitive test	Calculation of compound score
Processing speed	Trail Making Test A<br>Symbol-Digit Modalities Test	$Z_{TMT-A} \times -1 + Z_{SDMT}/2$
Executive function	Trail Making Test A<br>Trail Making Test B<br>Verbal Fluency Test (Animals and jobs)	$Z_{(TMT-B/TMT-A)} \times -1 + (Z_{VFT-A} + Z_{VFT-J})/2$
Memory	Digit span test (Forward and backward)<br>Rey's Auditory Verbal learning test (Immediate recall and delayed recall)<br>Rey–Osterrieth Complex Figure Test (Immediate recall and delayed recall)	$Z_{(DST-f + DST-b)} + (Z_{RAVLT-I} + Z_{RAVLT-D})/2 +(Z_{ROCF-I} + Z_{ROCF-D})/2$

Z, the Z transformed score; DST-f, Digit span forward test; DST-b, Digit span backward test; RAVLT-I, the Immediate recall of Rey's Auditory Verbal learning test; RAVLT-D, the delayed recall of Rey's Auditory Verbal learning test; ROCF-I, the Immediate recall of Rey–Osterrieth Complex Figure Test;

ROCF -D, the delayed recall of Rey–Osterrieth Complex Figure Test; TMT, Trail Making Test; SDMT, Symbol-Digit Modalities Test, VFT-A, Verbal Fluency Test (animals); VFT-A, Verbal Fluency Test (jobs)

MRI acquisition

Parameters for each sequence: 3D T1-weighted Magnetization Prepared 2 Rapid Acquisition Gradient Echoes (MP2RAGE): 0.85mm isotropic voxels, repetition time (TR) = 5500 ms, inversion time (TI1 and TI2) = 700 and 2500 ms, field of view (FOV) = 218×272mm^2, Flip Angle = 4; 3D multi-echo fast low-angle shot images (9 echoes): 0.85mm isotropic voxels, TR = 44ms, ΔTE = 4ms, FOV = 197×245mm^2, Flip angle = 20; 3D fluid-attenuated inversion recovery (FLAIR) image: 0.85mm isotropic voxels, TR = 5000ms, TE = 394 ms, TI = 1800ms, FOV = 163×272mm^2, Flip angle = 120; Multi-shell DWI using multi-band accelerated echo planar imaging (EPI): 99 diffusion-weighted directions (3 × b = 200, 6 × b = 500, 30 × b = 1,000, and 60 × b = 3,000s/mm2), 10 × b = 0 images, 1.7mm isotropic voxels, TR = 3220ms, TE = 74 ms, FOV = 221×221mm^2 , Flip angle = 90; one b=0 image with acquisition parameters equal to the previous b=0 images, but acquired in opposite phase-encoding direction.

Identification of connected cortex

The WMH masks derived from the FLAIR images were linearly registered to T1 images and then to diffusion images using Functional MRI of the brain linear image registration tool (FLIRT).[1,2] The white/grey matter boundaries were also linearly registered to the diffusion images. Probabilistic tractography was performed from the WMH masks to the white/grey matter boundaries in the diffusion spaces. This was done using the probtractx2 function from FSL with the following parameters: number of samples = 5000, number of steps per sample = 2000, step length in mm = 0.5, curvature threshold = 0.2.[3] The WM/GM matter boundary was additional used to constrain tractography. Resulting tracts were registered to T1 images using the inversed transformation matrix from T1 images to diffusion images generated in above registering steps. Regions where the tracts reached the white/grey matter boundaries were defined as the WMH-connected regions. To determine different levels of the connectivity probability between the cortex and the WMH lesion, the resulting WMH-connected regions were thresholded at different levels. Based a previously published study,[4-6] the threshold of the lowest level set at 3.08 x 10^{-5} percent of the total streamlines sent out from the seed

masks (5000 times per voxel). WMH-connected regions with the medium and high levels of connectivity probability were determined by gradually increasing the values of the threshold until the volumes of thresholded the WMH-connected regions reached 50% (medium level) or 25% (high level) of the volumes of the low-level WMH-connected regions, as used in previous similar studies.[7,8]

Results

Table e-2. Results on each cognitive task

Cognitive task	Raw score
DST	
Forward, median (IQR)	9.0 (7.0-10.0)
backward, median (IQR)	5.0 (4.0-7.0)
RAVLT	
Immediate recall, median (IQR)	22.0 (19.0-25.0)
Delayed recall, median (IQR)	7.0 (4.0-8.8)
ROCF (Delayed recall)	
Immediate recall, median (IQR)	21.0 (16.0-25.0)
Delayed recall, median (IQR)	20.0 (16.0-24.0)
TMT-A, median (IQR)	45.2 (36.3-63.0)
TMT-B, median (IQR)	103.0 (81.3-153.1)
SDMT, median (IQR)	41.0 (32.0-47.8)
VFT, median (IQR)	
Animals	20.0 (16.0-23.0)
Jobs	15.0 (11.0-18.0)

DST, Digit span forward test; RAVLT, Rey's Auditory Verbal learning test; ROCF, Rey–Osterrieth Complex Figure Test; TMT, Trail Making Test; SDMT, Symbol-Digit Modalities Test; VFT, Verbal Fluency Test.

Table e-3.the connectivity probabilities of WMH to different cortical regions at three connectivity levels

Location	Desikan-Killiany atlas	High level	Medium level	Low level
	superiorfrontal	**0.24888**	**0.53337**	0.838535
	caudalmiddlefrontal	0.13648	0.291265	0.63656
	rostralmiddlefrontal	0.20227	0.361045	0.70671
	frontalpole	**0.53674**	**0.8502**	**0.978905**
	parsopercularis	**0.25371**	0.47833	0.785365
Frontal	parsorbitalis	0.21318	**0.551245**	**0.880005**
	parstriangularis	**0.37015**	**0.577375**	**0.872145**
	lateralorbitofrontal	0.07688	0.186365	0.523905
	medialorbitofrontal	0.21972	0.3239	0.666365
	precentral	0.20678	0.374775	0.72977
	paracentral	0.142375	0.47744	**0.851065**
	postcentral	0.18307	0.250905	0.59474
	superiorparietal	0.170905	0.37492	0.714645
parietal	inferiorparietal	0.1724	0.23677	0.585205
	supramarginal	0.148655	0.22152	0.58727
	precuneus	0.158025	0.48017	**0.838675**
	bankssts	0.144465	0.192505	0.587905
	transversetemporal	0.020885	0.138355	0.539115
	superiortemporal	0.16161	0.397985	0.82154
Temporal	temporalpole	0.06608	0.3185	0.723
	entorhinal	0.06187	0.113065	0.413925
	middletemporal	0.142515	0.32702	0.767805
	inferiortemporal	0.142755	0.353315	0.749355
	fusiform	0.07921	0.20098	0.602855
	lateraloccipital	0.127265	0.24399	0.62291
occipital	cuneus	0.17711	0.446875	0.79251
	lingual	0.169145	0.3489	0.711615
	pericalcarine	**0.231805**	**0.53718**	0.814345
insula	insula	0.158465	0.39924	0.716645
	caudalanteriorcingulate	0.215755	0.513365	0.80609
	rostralanteriorcingulate	0.182145	0.28347	0.64236
limbic	posteriorcingulate	0.164535	0.401485	0.734815
	isthmuscingulate	0.205405	0.5194	0.825865
	parahippocampal	0.099665	0.275955	0.69001

Values in bold are the top-five ranking of cortical regions with highest connectivity probabilities to the WMH at each connectivity level.

Table e-4. Relation between normalized WMH volumes and the cortical thickness, R1, R2*, susceptibility values of the WMH-connected regions at three connectivity levels

	WMH volumes	
	β	*p-corrected value*
Cortical thickness (WMH-connected regions)		
Low level	-0.25	**0.006**
Medium level	-0.23	**0.046**
High level	-0.09	0.395
R1 values (WMH-connected regions)		
Low level	-0.05	0.632
Medium level	0.09	0.632
High level	0.14	0.510
R2* values (WMH-connected regions)		
Low level	-0.08	0.908
Medium level	0.09	0.836
High level	-0.01	0.908
Susceptibility values (WMH-connected regions)		
Low level	-0.04	0.846
Medium level	-0.05	0.846
High level	0.02	0.846

WMH, white matter hyperintensities; normalized WMH volumes, WMH volumes were normalized by the intracranial volume (ICV); bold values: *p-corrected* < 0.05, p values were corrected for multiple comparisons using *Hommel-Hochberg* method.

Table e-5. Relation between the cortical thickness, R1, R2*, susceptibility values of the WMH-connected regions and cognitive function at three connectivity levels (Model 2)

	Processing speed		Executive function		Memory	
	β	*p*-corrected value	β	*p*-corrected value	β	*p*-corrected value
Cortical Thickness of the WMH-connected regions						
High level	0.15	0.131	-0.09	0.946	-0.02	0.993
Medium level	0.20	0.131	0.06	0.946	0.04	0.993
Low level	0.30	0.131	0.36	0.154	0.10	0.993
R1 of the WMH-connected regions						
High level	0.18	**0.035**	0.06	0.946	0.04	0.993
Medium level	0.20	**0.018**	0.09	0.915	0.08	0.993
Low level	0.21	**0.030**	0.10	0.898	0.10	0.993
R2* of the WMH-connected regions						
High level	0.28	**0.014**	-0.14	0.800	0.01	0.993
Medium level	0.42	**0.002**	-0.26	0.253	0.02	0.993
Low level	0.37	**0.011**	-0.21	0.458	0.09	0.993
Susceptibility of WMH-connected regions						
High level	0.14	0.131	-0.04	0.946	0.00	0.993
Medium level	0.22	**0.025**	-0.02	0.946	0.00	0.993
Low level	0.25	**0.030**	0.01	0.946	-0.02	0.993

Model 2 is adjusted for age, education years, the areas of WMH-connected regions, the normalized WMH volumes, the mean cortical thickness or R1 or R2* or susceptibility values of the WMH-unconnected regions, and the mean MD values of the connecting tracts; WMH, white matter hyperintensities, MD, mean diffusivity; bold values: *p*-corrected < 0.05, *p* values were corrected for multiple comparisons using *Hommel-Hochberg* method.

Table e-6. Factor load and model fit indicators of the latent variables using confirmatory factor analysis.

	Latent variable 1 (WMH-connected regions)	Latent variable 2 (WMH-unconnected regions)
Standardized factor load		
Cortical thickness	0.490	0.066
R1 values	0.955	0.590
R2* values	0.595	0.766
Susceptibility values	0.862	0.364
Model fit indicators		
CFI	0.999	>0.999
RMSEA	0.035	<0.001
SRMR	0.020	0.021

Latent variable 1, representing global cortical abnormalities of WMH-connected; Latent variable 2, representing global cortical abnormalities of WMH-unconnected; WMH-connected and WMH-unconnected regions were identified at the high connectivity level. WMH, white matter hyperintensities; CFI, Comparative Fit Index; RMSEA, Root Mean Square Error of Approximation; SRMR, Standardized Root Mean Residual.

Table e-7. Relation between MD of the connecting tracts and the cortical thickness, R1, R2*, susceptibility values of the WMH-unconnected regions at three connectivity levels

	MD of the connecting tracts	
	β	*p-corrected value*
Cortical thickness (WMH-unconnected regions)		
Low level	-0.00	0.997
Medium level	-0.03	0.997
High level	-0.05	0.959
R1 values (WMH-unconnected regions)		
Low level	-0.08	0.392
Medium level	-0.06	0.392
High level	-0.06	0.392
R2* values (WMH-unconnected regions)		
Low level	-0.15	0.060
Medium level	-0.16	0.062
High level	-0.13	0.064
Susceptibility values (WMH-unconnected regions)		
Low level	0.10	0.201
Medium level	0.04	**0.018**
High level	0.21	**0.006**

WMH, white matter hyperintensities; MD, mean diffusivity; bold values: *p*-corrected < 0.05, *p* values were corrected for multiple comparisons using *Hommel-Hochberg* method.

Table e-8. Relation between normalized WMH volumes and the cortical thickness, R1, R2*, susceptibility values of the WMH-unconnected at three connectivity levels

	Normalized WMH volumes	
	β	*p-corrected value*
Cortical thickness (WMH-unconnected regions)		
Low level	-0.23	**0.012**
Medium level	-0.15	0.057
High level	-0.15	0.057
R1 values (WMH-unconnected regions)		
Low level	-0.07	0.926
Medium level	-0.05	0.926
High level	-0.01	0.926
R2* values (WMH-unconnected regions)		
Low level	-0.14	0.480
Medium level	-0.05	0.652
High level	-0.06	0.652
Susceptibility values (WMH-unconnected regions)		
Low level	-0.19	0.189
Medium level	-0.05	0.622
High level	-0.05	0.622

WMH, white matter hyperintensities, normalized WMH volumes, WMH volumes were normalized by the intracranial volume (ICV); bold values: *p*-corrected < 0.05, p values were corrected for multiple comparisons using *Hommel-Hochberg* method.

Table e-9. Relation between MD of the connecting tracts, normalized WMH volumes and the R1, R2*, susceptibility values of the WMH-connected regions while adjusting for cortical thickness at three connectivity levels.

	Normalized WMH volumes		MD of the connecting tracts	
	β	*p-corrected*	β	*p-corrected*
R1 values (WMH-connected regions)				
Low	-0.06	0.559	-0.14	0.069
Medium	0.09	0.559	-0.12	0.069
High	0.13	0.438	-0.11	0.069
R2* values (WMH-connected regions)				
Low	-0.01	0.937	-0.15	**0.031**
Medium	0.11	0.937	-0.22	**0.008**
High	0.01	0.937	-0.29	**<0.001**
Susceptibility values (WMH-connected regions)				
Low	-0.03	0.763	-0.02	0.792
Medium	-0.02	0.763	-0.10	0.142
High	0.04	0.763	-0.15	**0.042**

WMH, white matter hyperintensities, normalized WMH volumes, WMH volumes were normalized by the intracranial volume (ICV); MD, mean diffusivity; bold values: *p*-corrected < 0.05, p values were corrected for multiple comparisons using *Hommel-Hochberg* method.

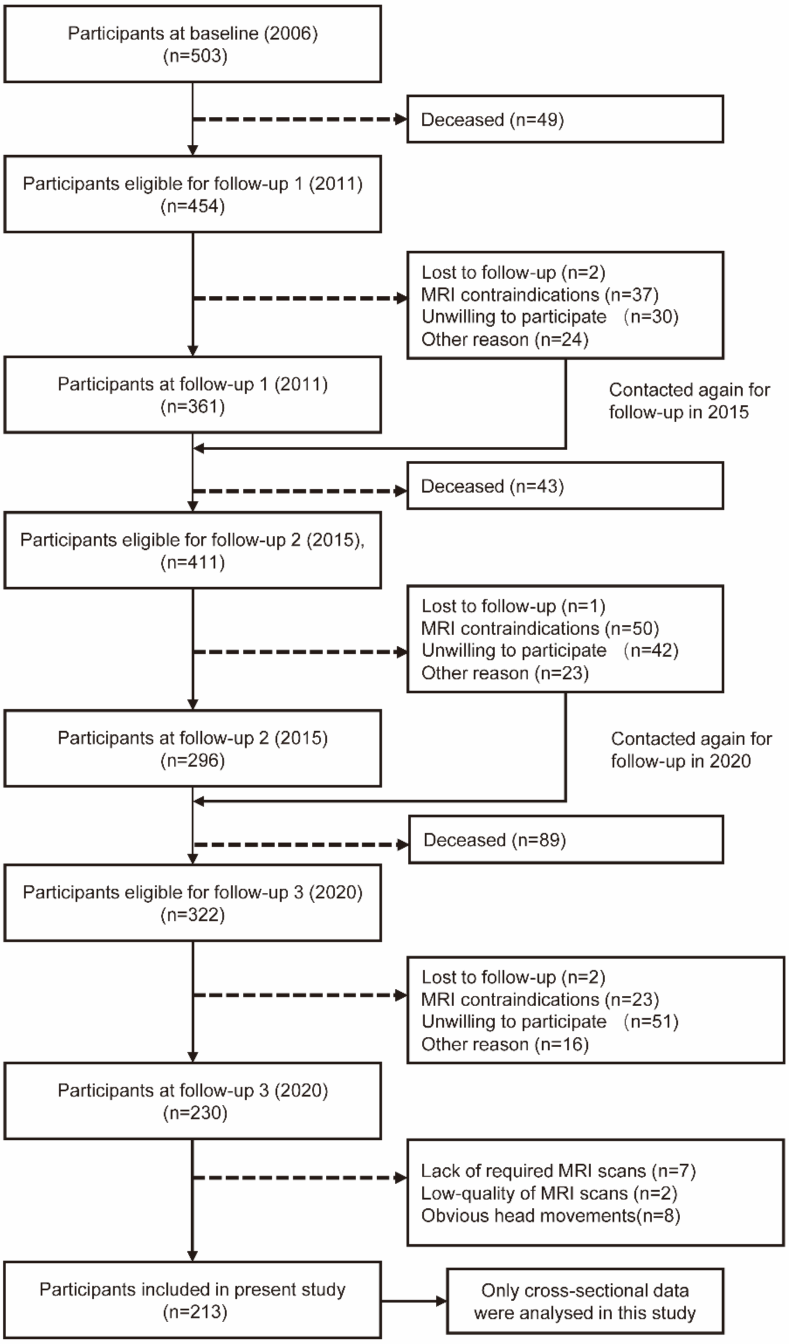

Figure e-1. Flow chart of the present study.

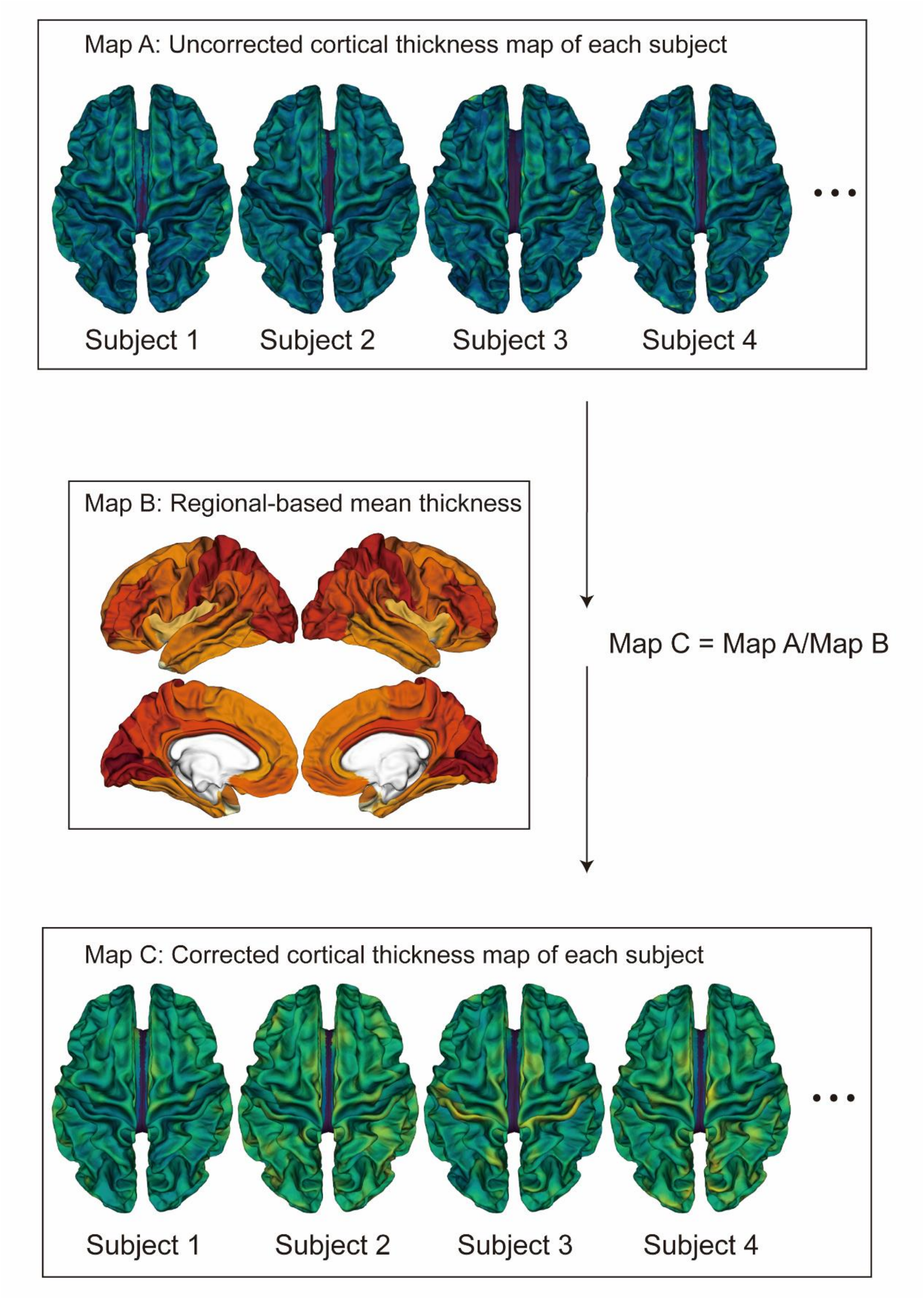

Figure e-2 Schematic diagram of correcting the inter-regional differences in cortical thickness. Similar steps were performed in the surface-based R1, R2* and susceptibility maps of each participant to correct the inter-regional differences in myelin and iron.

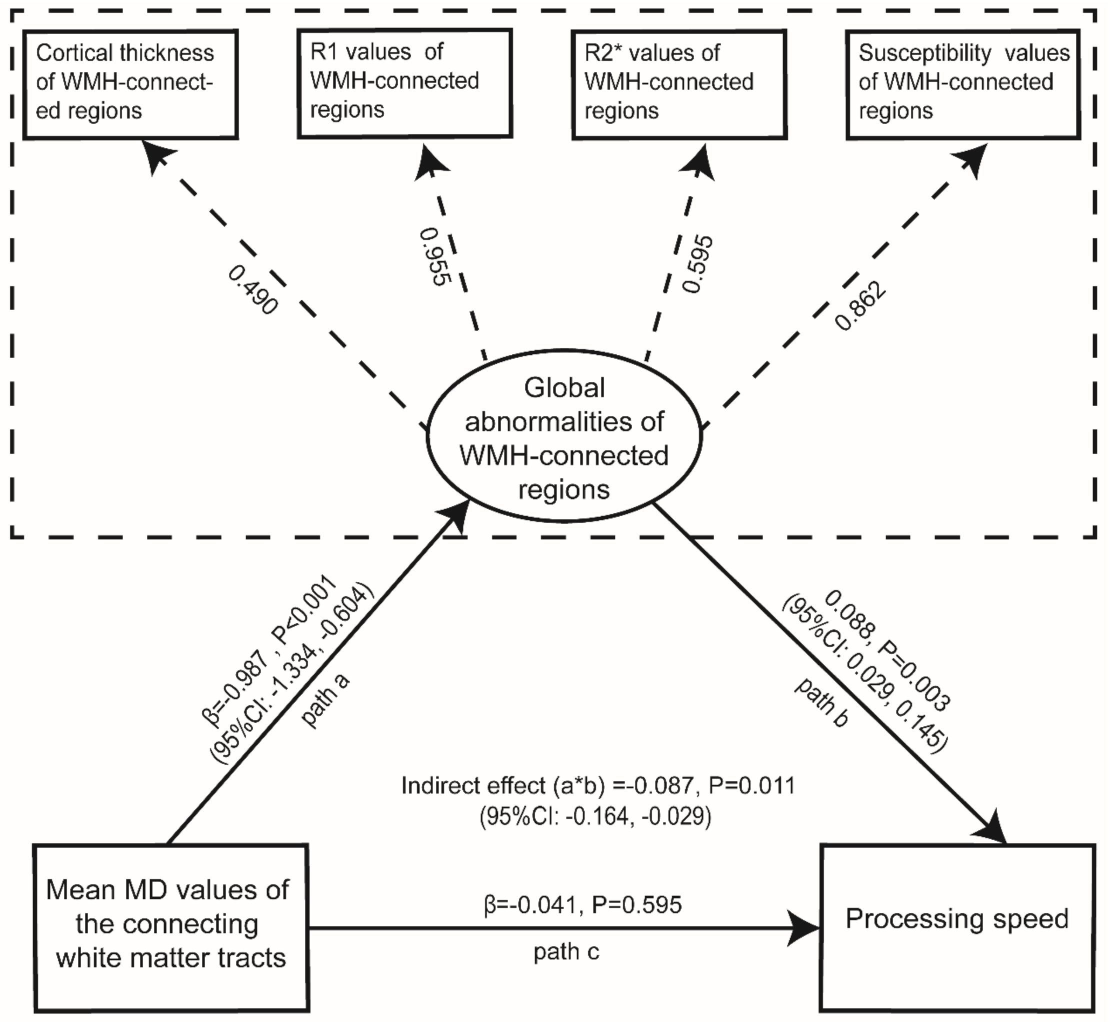

Figure e-3. Structural equation model for the relations between the microstructural damage of the connecting white matter tract, the abnormalities of WMH-connected regions and cognitive performances. WMH-connected regions were identified at the high-connectivity level. Covariances and control variables were omitted for readability. Upper panel: the confirmatory factor analysis (CFA) used to fit the latent variable representing global cortical abnormalities of the WMH-connected regions, the values attached to each dashed arrow are standardized factor load of each cortical measurements on the constructed latent variable; Lower panel: the mediation analysis, of mean MD values of the connecting white matter tract, the global cortical abnormalities of the WMH-connected regions and processing speed, path a: relationship between mean MD values of the connecting white matter tract and global cortical abnormalities of the WMH-connected regions, path b: relationship between global cortical abnormalities of the WMH-connected regions and processing speed. path c: direct relationship between mean MD values of the connecting white matter tract and processing speed. MD, mean diffusivity; WMH, white matter hyperintensities; 95%CI, 95% confidence interval.

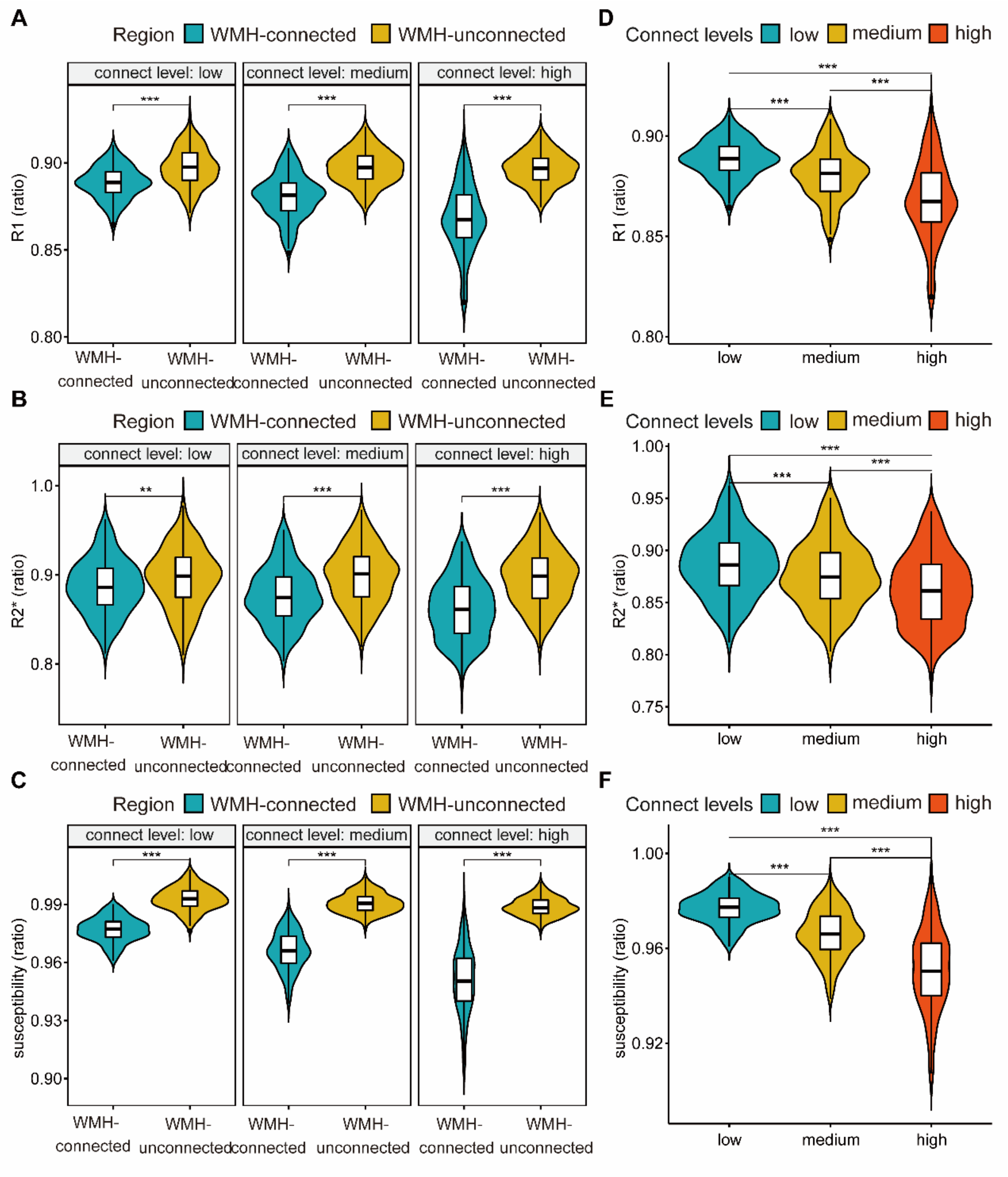

Figure e-4. Comparison of R1, R2*, susceptibility values between WMH-connected regions and WMH-unconnected regions (A-C), and between different connectivity levels (D-F) while adjusting cortical thickness. **, *p-corrected* < 0.01, ***, *p-corrected* < 0.001

Reference

1. Jenkinson M, Smith S. A global optimisation method for robust affine registration of brain images. *Medical image analysis*. Jun 2001;5(2):143-56. doi:10.1016/s1361-8415(01)00036-6
2. Jenkinson M, Bannister P, Brady M, Smith S. Improved optimization for the robust and accurate linear registration and motion correction of brain images. *NeuroImage*. Oct 2002;17(2):825-41. doi:10.1016/s1053-8119(02)91132-8
3. Behrens TE, Berg HJ, Jbabdi S, Rushworth MF, Woolrich MW. Probabilistic diffusion tractography with multiple fibre orientations: What can we gain? *NeuroImage*. Jan 1 2007;34(1):144-55. doi:10.1016/j.neuroimage.2006.09.018
4. Rilling JK, Glasser MF, Preuss TM, et al. The evolution of the arcuate fasciculus revealed with comparative DTI. *Nature neuroscience*. Apr 2008;11(4):426-8. doi:10.1038/nn2072
5. Zhuo J, Fan L, Liu Y, Zhang Y, Yu C, Jiang T. Connectivity Profiles Reveal a Transition Subarea in the Parahippocampal Region That Integrates the Anterior Temporal-Posterior Medial Systems. *The Journal of neuroscience : the official journal of the Society for Neuroscience*. Mar 2 2016;36(9):2782-95. doi:10.1523/jneurosci.1975-15.2016
6. Seifert R, Telli T, Opitz M, et al. Unspecific (18)F-PSMA-1007 Bone Uptake Evaluated Through PSMA-11 PET, Bone Scanning, and MRI Triple Validation in Patients with Biochemical Recurrence of Prostate Cancer. *Journal of nuclear medicine : official publication, Society of Nuclear Medicine*. May 2023;64(5):738-743. doi:10.2967/jnumed.118.215434
7. Duering M, Righart R, Csanadi E, et al. Incident subcortical infarcts induce focal thinning in connected cortical regions. *Neurology*. Nov 13 2012;79(20):2025-8. doi:10.1212/WNL.0b013e3182749f39
8. Duering M, Righart R, Wollenweber FA, Zietemann V, Gesierich B, Dichgans M. Acute infarcts cause focal thinning in remote cortex via degeneration of connecting fiber tracts. *Neurology*. Apr 21 2015;84(16):1685-92. doi:10.1212/wnl.0000000000001502
