# Supplementary figures and images for: Boost of innate immunity cytokines as biomarkers of response to extracorporeal photopheresis in patients with leukaemic cutaneous T-cell lymphoma
Source: Br J Dermatol. 2023 Jul 6;189(5):603–11. doi: 10.1093/bjd/ljad220 (PMC13077219; doi:10.1093/bjd/ljad220)

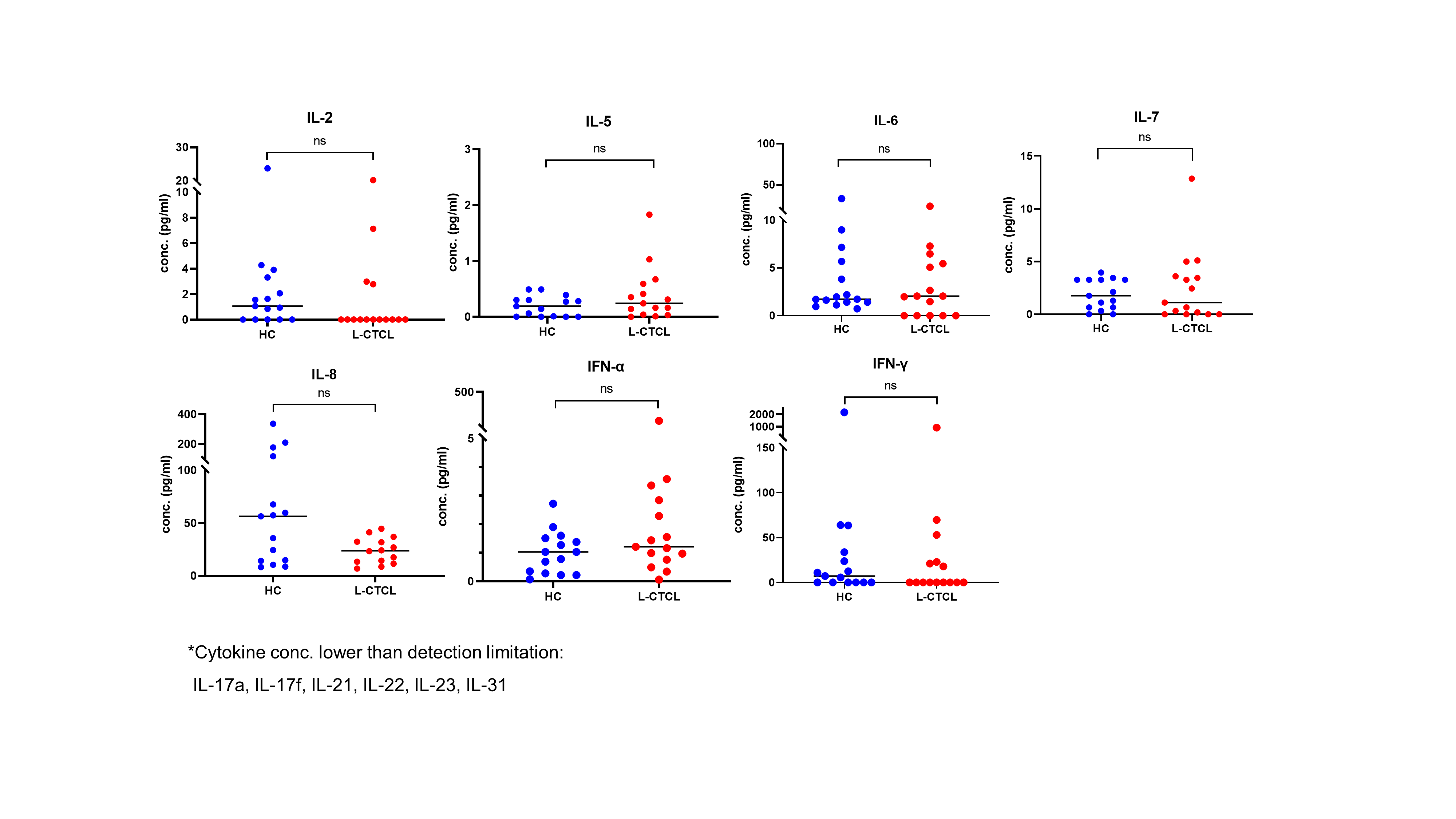

Supplement: ljad220_Supplementary_Data [file ljad220_supplementary_data.zip › ECP cytokine paper_suppl figure 1.TIF]
